# Qoppa as a New Pan-Tumor Synthetic Parameter Derived from Tumor-Associated Biomarkers for Identifying Oncology Patients at High Risk of Metastasis: A Prospective Pilot Study

**DOI:** 10.3390/jcm15020846

**Published:** 2026-01-20

**Authors:** Javier Diaz-Santos, Alba Rodriguez-Valle, Beatriz Berrocal-Gavilan, Olivia Urquizar-Rodriguez, Silvia Montoro-Garcia

**Affiliations:** 1 HM CIOCC MALAGA (Centro Integral Oncológico Clara Campal), Hospital Internacional HM Santa Elena, 29620 Málaga, Spain; amrodriguezvalle@hmhospitales.com (A.R.-V.); bberrocalg@hmhospitales.com (B.B.-G.); ourquizar@hmhospitales.com (O.U.-R.); 2 Instituto de Investigación Sanitaria HM Hospitales, 28050 Madrid, Spain; 3 Preclinical Research of Bioactive Compounds and Drugs (PREBIOF), Izpisúa Lab HiTech, Faculty of Health Sciences, Universidad Católica de Murcia (UCAM), Campus los Jerónimos, 30107 Murcia, Spain; smontoro@ucam.edu

**Keywords:** liquid biopsy, biomarkers, metastasis, precision medicine, disease progression

## Abstract

**Background/Objective:** Early detection of metastatic progression remains a major challenge in precision oncology. Conventional radiological imaging cannot reliably identify micrometastatic disease. Although circulating tumor DNA is promising for minimal residual disease detection, organ-derived response biomarkers reflecting tissue adaptation to secreted factors remain unexplored. We hypothesized that integrating such biomarkers with global laboratory parameters would generate a synthetic variable with improved discrimination for de novo metastasis and mortality. **Methods:** This prospective observational pilot study enrolled 30 patients (median age 64.4 years; 56.7% female) with heterogeneous solid malignancies. Peripheral blood biomarkers responsive to tumor-secreted soluble factors (*n* = 11) were quantified using a multiplexed beads Luminex immunoassay. Global analytical parameters (*n* = 20) were derived from routine laboratory assessments. Hierarchical agglomerative clustering analysis generated two synthetic variables: Stigma (Ϛ) and Qoppa (Ϙ). Receiver operating characteristic curve analysis, Kaplan–Meier survival analysis, and Cox regression were used to evaluate the performance. **Results:** Qoppa demonstrated acceptable discriminatory performance for de novo metastasis (AUC = 0.78). For mortality prediction, performance varied by disease status (overall AUC = 0.78): superior in non-metastatic patients (AUC = 0.98) but negligible in those with baseline metastases. Kaplan–Meier analysis confirmed significant survival differences (*p* = 0.042 overall survival; *p* = 0.024 for metastasis-free survival in the non-metastatic subgroup). Differences in biomarker expression and clinical variables (stage, tumor burden, and metastatic burden) were observed between the high and low Qoppa strata. **Conclusions:** In this small heterogeneous pilot cohort, Qoppa provides a proof of concept that integrating organ-derived response biomarkers with routine laboratory parameters may capture clinically relevant signals for metastatic risk stratification in oncology patients. This composite parameter supports the generation of hypotheses for future biomarker-driven research and clinical test development. External validation in larger multicenter cohorts is required before clinical implementation.

## 1. Introduction

The early detection of metastatic progression is one of the most important clinical challenges in oncology. Metastases are responsible for more than 90% of mortality in solid tumors, with hepatic, bone, pulmonary, and cerebral localizations being the most frequent, at an approximate incidence of 8–22 cases per year per 100,000 inhabitants [1,2,3,4,5]. Despite therapeutic advances, diagnosis at the metastatic stage implies a worse prognosis as an independent factor, particularly grave in the case of synchronous metastases at primary tumor diagnosis [2,4].

Conventional metastasis detection is performed using radiological imaging techniques, which present important limitations in identifying micrometastatic disease. These techniques require a minimum tumor volume for visualization and do not allow continuous non-invasive monitoring during clinical follow-up [6,7]. Therefore, the search for non-invasive biomarkers that enable the early detection of metastatic risk has become a priority area of research in precision oncology [8,9,10,11]. Biomarkers can enable the identification of high-risk patient subgroups, appropriate therapeutic stratification, and monitoring of therapeutic response with higher sensitivity than conventional methods [6,7,8,9].

Primary tumors orchestrate metastatic dissemination through organ-specific premetastatic niche formation [1,12,13,14,15,16,17,18]. This process is driven by tumor-secreted soluble factors and exosomes that trigger immunoinflammatory remodeling in distant organs [13,14,15], particularly those with high capillary density, such as liver, lung, and brain [17,18]. These organ-derived pathophysiological responses to tumor-secreted soluble factors are reflected through circulating biomarkers [19,20], whereas systemic biological responses to tumor activity are captured by global laboratory parameters. These parameters encompass, among others, the neutrophil-to-lymphocyte ratio, systemic inflammation indices, and acute phase reactants, which may serve as complementary prognostic markers alongside response biomarkers [21,22,23,24,25].

While circulating tumor DNA (ctDNA) liquid biopsy demonstrates clinical utility for minimal residual disease detection—with lead times of 10.5 months for recurrence detection [26,27,28]—this approach remains tumor-centric and fails to capture the organ microenvironmental adaptation essential for metastatic establishment. Organ-derived response biomarkers reflecting tissue-specific adaptation to tumor-secreted soluble factors remain poorly explored, despite offering complementary information about the microenvironmental conditions that predispose target organs to metastatic disease. The integration of both tumor molecular characteristics and organ-specific biomarkers captures the complex biology of tumor–host interactions essential for disease prevention [29]. To our knowledge, no clinical analysis has systematically evaluated these organ-derived biomarkers, representing a significant knowledge gap.

We hypothesized that response biomarkers to tumor-secreted soluble factors detectable in peripheral blood, when integrated with established global laboratory parameters, would generate a predictive synthetic variable with enhanced discriminatory capacity for de novo metastasis risk and mortality compared to individual parameters in isolation.

The preliminary results of this pilot study were presented as a poster at the 3rd International Online Conference on Clinical Medicine (IOCCM 2025) [30]. This manuscript provides the complete dataset, detailed methodology, and extended survival analysis not previously published.

## 2. Materials and Methods

### 2.1. Study Population

A prospective observational pilot study was conducted, and written informed consent was obtained from each patient. The study population was actively recruited at the Clara Campal Comprehensive Oncology Center of HM Hospitales in Málaga (HM CIOCC, Málaga, Spain) between 1 May 2024 and 15 July 2025. A prospective design was selected to enable systematic biomarker sample collection without dependence on a pre-existing biobank.

### 2.2. Study Design

Eligible participants included patients with a histologically confirmed diagnosis of one or more malignancies, with the requirement that at least one primary neoplasm was a solid tumor rather than a hematologic malignancy (lymphoma, leukemia, or myeloma). Participants (>18 years old) provided informed consent prior to their enrolment in the study. Patients were diagnosed, treated, or monitored at the Clara Campal Comprehensive Oncology Center of HM Hospitales in Málaga (HM CIOCC Málaga). Conversely, patients with temporary or permanent inability to provide informed consent owing to cognitive impairment, linguistic barriers, or other factors were excluded from the study. Additionally, individuals who requested verbal consent alone without formal written documentation were excluded in accordance with institutional ethical requirements and good clinical practice guidelines.

Given the exploratory nature of this pilot study, a sample size of approximately 5% of our available oncology patient pool (approximately 500 patients) was targeted, resulting in an estimated sample size of 25 patients.

### 2.3. Biomarker Selection

Biomarker selection was performed through a review of the published literature, seeking preclinical and clinical evidence of molecules secreted by specific organs (liver, lung, blood–brain barrier) in response to tumor-secreted soluble factors, possessing sufficient tissue specificity to enable organ-specific inferences, demonstrating clinical variability in high tumor burden contexts, and measurable by available technical methods. The eleven selected plasmatic biomarkers are listed in Table 1.

Global analytical parameters were selected based on their association with oncological prognosis and metastatic risk. These parameters are derived from routine laboratory assessments available in standard clinical practice, thereby enhancing the applicability of the strategy. Twenty qualitative and quantitative analytical parameters were selected, including complete blood count values, prognostic indices, metabolic parameters, hepatorenal function markers, inflammatory markers, and hemostatic indices. In what follows, to differentiate them from response biomarkers, the aliases for the global laboratory parameters are written in lowercase. The parameters are listed in Table 2.

### 2.4. Blood Sampling and Biochemical Determinations

The determination of response biomarkers to tumor-secreted soluble factors was performed in EDTA peripheral blood obtained after fasting through a customized multiplex immunoassay (Merck Millipore, Burlington, MA, USA), following the manufacturer’s instructions. Multiplex utilizes polystyrene bead-based technology to measure the eleven markers. Based on the measurements of standard concentrations provided by the manufacturer, standard curves were utilized to convert optical density values into concentrations (pg/mL). Supernatant samples were thawed once and clarified by centrifugation at 10,000× *g* for 10 min. Next, the plate was loaded into the Luminex 200 system (Luminex Corporation, Austin, TX, USA) for reading. Analytes were measured in duplicate across three plates/panels: HAGE1MAG-20K-6p (FGF21, GDF15, IL6, IL10, IL18, leptin), HLPPMAG-57K-2p (ANGPTL4, HGF), and HNDG3MAG-36K-3p (cathepsin-D, ICAM1, MPO). Based on the measurements of the standard concentrations provided by the manufacturer, standard curves were utilized to convert the median fluorescence intensity (MFI) values into concentrations (pg/mL or ng/mL). Across quantifiable measurements, the median intra-assay CV was 7.84%, and 80% of measurements showed CV ≤ 10%. The operational limit of detection (LOD) for each analyte was defined as the expected concentration for the lower standard concentration. To avoid over-detection bias that would artificially inflate the results, missing values were imputed using the minimum detected value within the assay range for each parameter. In parallel, global laboratory parameters were collected, calculated from routine laboratory studies performed prior to plasma extraction. All routine laboratory studies were conducted exclusively within the study period. The sample collection and laboratory data extraction served as baseline covariables, enabling prospective determination of the response biomarkers and global laboratory parameters. The clinical follow-up was extended to 15 July 2025, permitting evaluation of clinical events, including metastatic progression, de novo metastasis, and mortality.

### 2.5. Statistical Analyses

The clinical data were systematically collected at four defined assessment timepoints: diagnosis, biomarker sampling, post-sampling, and end of follow-up. The post-sampling period was defined as the interval from biomarker sampling to the next occurrence of disease progression, patient death, or the end of follow-up. The documented clinical variables included histological diagnosis, prior or concurrent malignancies, disease stage, locoregional lymph node involvement, primary tumor volume, and temporal intervals between major clinical events. A description of the disease burden determination is available in the Supplementary Materials.

The data analysis was structured according to the following hierarchical framework: descriptive statistical analysis of demographic and clinical characteristics; hierarchical agglomerative clustering analysis to identify patient clusters based on biomarker and analytical parameter profiles; generation of synthetic cluster-derived variables capturing integrative biomarker and analytical information for each identified cluster; comparative analysis of analytical and clinical differences between high and low synthetic cluster variable strata; and survival analyses encompassing both overall survival and metastasis-free survival outcomes.

The descriptive statistical analysis of the entire study population was performed with quantitative variables expressed as the mean or median and range according to the distribution characteristics. The Shapiro–Wilk test was used to evaluate the normality of the biomarkers and global laboratory parameters. Variables failing normality testing informed the selection of subsequent non-parametric statistical approaches. Hence, all biomarkers and global analytical parameters were normalized using min–max scaling.

Hierarchical agglomerative clustering analysis was performed using the Euclidean distance as the similarity metric and Ward’s minimum variance method for linkage. The determination of optimal cluster number was determined using two complementary approaches: the elbow method and silhouette score analysis. Dendrograms and heat maps were generated to visualize the cluster architecture and relationships between biomarkers and analytical parameters.

A novel synthetic variable was derived for each identified cluster by weighted summation of the min–max scaled variables according to the following equation:

(1) $$y_{i}=\sum_{j=1}^{n}W_{j}x_{ji}+\sum_{k=1}^{m}S_{k}z_{ki},$$

where $y_{i}$ represents the synthetic cluster variable, $x_{ji}$ and $z_{ki}$ are the min–max scaled global laboratory parameters and response biomarkers, respectively, and $W_{j}$ and $S_{k}$ are the assigned weights. Given the pilot nature of this study and insufficient prior knowledge to justify differential weighting, uniform unit weights were employed ($W_{j}=1,$ and $S_{k}$ = 1 for all *j* and *k*). This approach avoids subjective bias and prevents overfitting in small datasets, where data-driven weight optimization risks fitting noise rather than signal [74,75]. Consequently, the synthetic cluster variable is mathematically equivalent to the sum of all the scaled variables included in that cluster.

To assess the discriminatory capacity of synthetic cluster-derived variables, the study population was stratified into two cohorts: patients with existing metastases at biomarker sampling and patients without existing metastases at biomarker sampling. Receiver operating characteristic (ROC) curves were generated for each synthetic cluster-derived variable in relation to mortality risk across the entire study population and within each stratified cohort. Additionally, ROC curves were constructed to evaluate the capacity of each synthetic variable to predict post-sampling de novo metastases’ appearance in the non-metastatic population and post-sampling metastatic disease progression in the metastatic population. From these ROC curves, the optimal cutoff thresholds were determined for each synthetic cluster-derived variable. Furthermore, the individual discriminatory contributions of the biomarker component and the global laboratory parameter components within each synthetic cluster variable were separately analyzed for each clinical event. Although patient monitoring continued beyond the first post-sampling event, with the potential for disease stabilization or subsequent progression, the ROC analysis focused exclusively on the first documented post-sampling event. This ensures methodological clarity and facilitates the accurate characterization of the classifier role of the synthetic cluster variables. The mortality was determined by official death certification. De novo metastasis was defined as the weighted metastatic burden increasing from zero to >0 in baseline metastasis-free patients. Metastatic progression was defined as a positive increment in the weighted metastatic burden from baseline to follow-up in patients with baseline metastases. All determinations were performed in duplicate by a single investigator. For each ROC curve, optimism correction was also performed using the bootstrap of the area under the curve (AUC) with 1000 bootstraps per case. The ninety-five percent confidence intervals (95% CI) were obtained for both the area under the curve and the optimal cutoff point. Furthermore, the *p*-value for the comparison was calculated using the Mann–Whitney U test for each ROC curve versus the absence of discriminatory capacity (AUC = 0.5).

For biomarkers and laboratory parameters with a non-normal distribution, the Mann–Whitney U test was employed to compare biomarkers and analytical parameters between high- and low-level synthetic cluster-derived variables. Student’s t-test was used for normally distributed parameters.

Clinical variables, including the stage at diagnosis, stage at biomarker sampling, post-sampling stage, tumor burden metrics, weighted metastatic burden, and the presence of de novo metastases following sampling, were compared between the high and low synthetic cluster-derived variable groups. For that purpose, the Mann–Whitney U test was applied for clinical variables with non-normal distribution, and for clinical variables with normal distribution, the t-Student test was used.

Kaplan–Meier survival analysis was performed in the non-metastatic population at the time of biomarker sampling, stratifying patients by synthetic cluster-derived variable level (high vs. low). Overall survival (OS) and metastasis-free survival (MFS) curves were generated, also stratifying patients by synthetic cluster-derived variable level (high vs. low). Multivariate Cox proportional hazards regression analysis was used to analyze Qoppa as the principal independent variable, adjusting for the age, sex, histological type, and TNM stage. Hazard ratios (HR) with ninety-five percent confidence intervals were reported.

## 3. Results

Thirty patients with cancer were recruited (median age 64.4 years, range 32.3–79.5 years; 17 women 56.7%, 13 men 43.3%). Thirteen patients had a family history of cancer, which was limited to only nine patients in the case of first-degree relatives. Three patients (10%) had a history of successfully treated malignancy, and no cases of concurrent or metachronous neoplasms were documented. Among the comorbidities, two patients had long-standing rheumatoid arthritis requiring chronic immunomodulation, and one was HIV-seropositive with virological control on highly active antiretroviral therapy; no limiting disease-related sequelae were observed in these three patients.

The primary malignancies were heterogeneous, with breast cancer predominating (23.3%), followed by lung cancer (13.3%), and colon and bladder cancers equally represented (10% each). Histologically, adenocarcinoma was the most frequent subtype (26.7%), followed by infiltrating ductal carcinoma (16.7%), neuroendocrine carcinoma (13.3%), and urothelial carcinoma (13.3%). At diagnosis, the median disease stage was 2–3, with locoregional lymph node involvement in 36.7% of patients and metastatic disease in 23.3%. Among patients with metastasis at diagnosis, hepatic involvement was the most common (71.4%), followed by bone (57.1%), peritoneal (42.9%), and pulmonary metastases (14.3%). The equivalent ellipsoidal primary tumor volume showed substantial heterogeneity (median 6481.89 mm^3^, range 65.45–1,518,661.60 mm^3^), with a tumor burden at diagnosis ranging from 1 to 13 (median 1, mean 2.8). A concise overview of the metastatic status at sample collection is available in the Supplementary Materials.

At the time of biomarker sampling, 60% of the patients had active disease (median stage 3–4, a median tumor burden of 2, and a range between 0 and 14). The median disease duration from diagnosis to biomarker sampling was 0.39 years (range, 0–5.4 years), during which patients received a median of 1.5 treatment lines (range, 0–5). The median number of disease relapses prior to biomarker collection was zero (mean 0.4, range 0–2). The median follow-up duration was 202 days (IQR: 144.25–232.75). Seventeen patients had received prior oncologic treatment before sample collection, while 13 were treatment-naïve at that time. A summary of the values of the detected biomarkers, follow-up times, and prior treatments is provided in the Supplementary Material.

Post-sampling, metastatic disease progression was observed in 10 patients, disease stabilization in 11 patients, and a complete response in 9 patients. Both patients with stable disease and those with complete response remained in the same condition until the end of the follow-up. Among the ten patients with disease progression, two achieved disease stabilization by the end of follow-up, whereas eight experienced further disease progression despite additional lines of treatment. Of these eight patients, seven ultimately died. The main characteristics of the study population are shown in Table 3.

**Figure 1:**
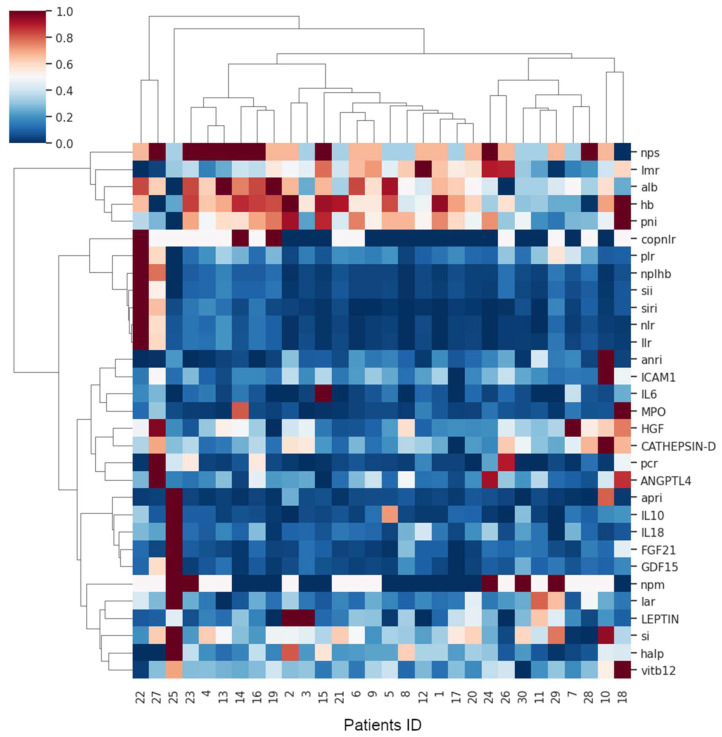
Hierarchically clustered heat map of variables (vertical axis) and patients (horizontal axis) across the complete study population. It was performed using variable aggregation and the Euclidean distance as the similarity metric, with Ward’s minimum variance method for linkage.

**Figure 2:**
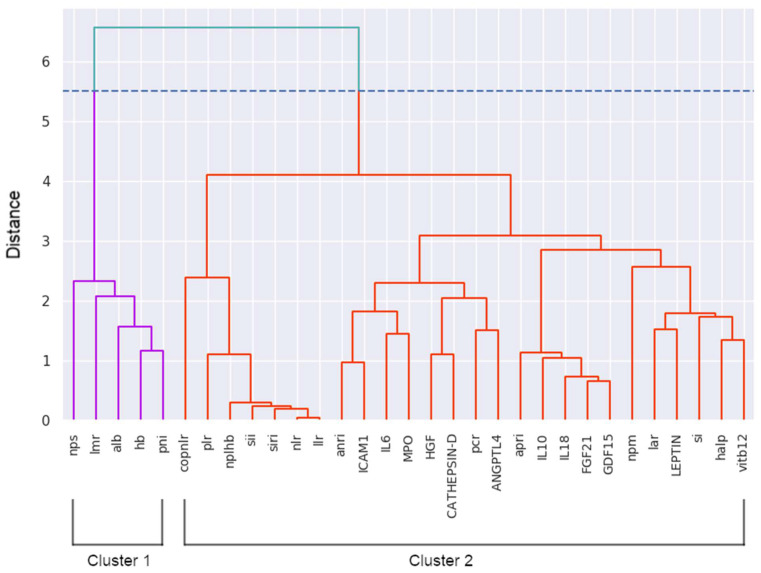
Hierarchical cluster dendrogram by variable for the complete study population. The optimal number of clusters was two, according to the elbow method and silhouette score analysis. The cutoff point is indicated by a blue dashed line. Cluster 1 variables are shown as blue branches and cluster 2 variables as red branches in the dendrogram.

Hierarchical clustering analysis using variable aggregation for the entire study population revealed the grouping of variables shown in the heat map in Figure 1. According to the elbow method and silhouette score, the variable set was divided into two well-defined hierarchical clusters. The first cluster included five variables, all of which were global parameters. The second cluster comprised all the response biomarkers and the remaining global laboratory parameters. The compositions of these clusters are shown in Figure 2.

For all global laboratory parameters and response biomarkers, individualized min–max scaling was performed per variable. Subsequently, for each of the two identified clusters, a synthetic study variable was created, with the resulting value for each patient corresponding to the sum of the scaled values of the individual variable values within each cluster. Accordingly, a cluster-derived variable named Stigma (Ϛ) was obtained from the first cluster, and another variable named Qoppa (Ϙ) was obtained from the second cluster. The contributions of global laboratory parameters and biomarkers to Qoppa are called Ϙ_G_ and Ϙ_B_, respectively; thus, Ϙ = Ϙ_G_ + Ϙ_B_. Because Stigma originated from a cluster comprising five variables and Qoppa from a cluster composed of 26 variables, their values in this study ranged from 0 to 5 for Stigma and from 0 to 26 for Qoppa. The extrapolation of these analytical results to subsequent studies does not imply that Stigma and Qoppa must be restricted to these ranges, as they may have higher values in different populations. The composition of Stigma (Ϛ) and Qoppa (Ϙ) is shown in Table 4. The profiles of the values of these variables are available in the Supplementary Material.

The assessment of these variables, Stigma and Qoppa, as classifiers for the risk of mortality, development of de novo metastasis in non-metastatic patients at baseline, and metastatic progression in metastatic patients at baseline was performed using receiver operating characteristic (ROC) curves. For all Stigma and Qoppa case studies, optimism correction was additionally performed via bootstrapping of the area under the curve using 1000 bootstraps per case.

**Figure 3:**
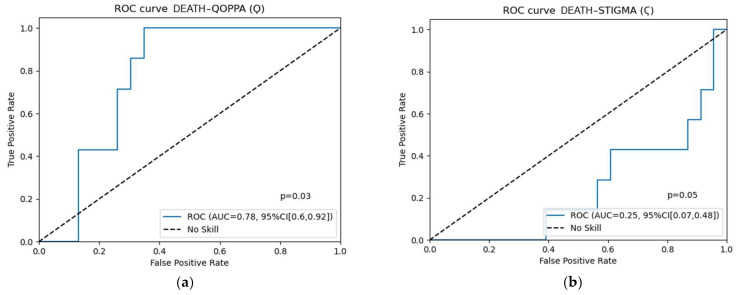

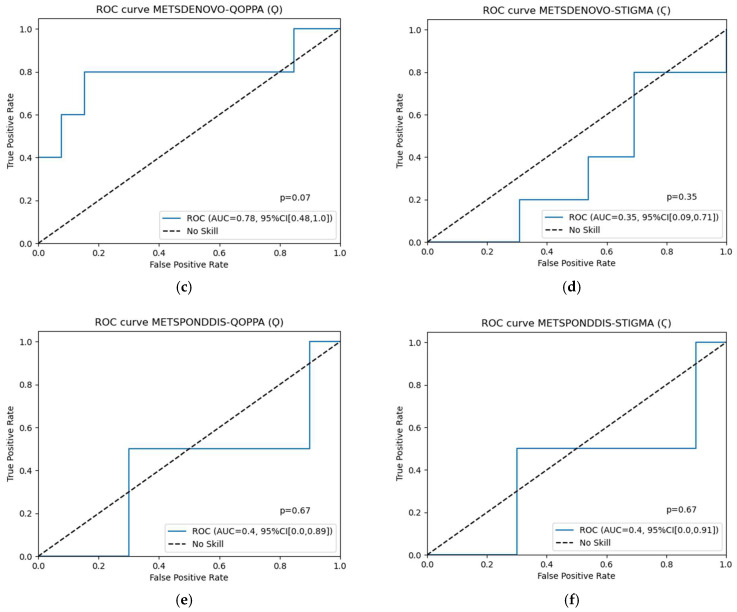
ROC curve analysis study of the role of Qoppa and Stigma as a classifier: (**a**,**b**) case study: death; (**c**,**d**) case study: development of metastasis de novo in patients with no metastasis at sample collection; (**e**,**f**) case study: metastasis progression in patients with metastasis at sample collection. A satisfactory role as classifier was observed in the figures (**a**): Qoppa and death and (**c**): Qoppa and new metastases’ development in the population without metastases at the time of sampling. For each case, the 95% confidence interval and *p*-value for comparing the ROC curve against no discriminatory ability (AUC = 0.5) using the Mann–Whitney U test are also shown.

The calculation of the area under the curve (AUC) identified Qoppa as an acceptable classifier with AUC = 0.78 for both mortality risk (95% CI: 0.60–0.92) and de novo metastasis (95% CI: 0.48–1). The optimism correction bootstrapping resulted in minimal variations for Qoppa as a classifier of death and de novo metastasis (AUC from the original 0.78 to the corrected 0.77). The AUC *p*-value for the ROC curves versus the null hypothesis of no discriminative ability (AUC = 0.5), using the Mann–Whitney U test, was 0.03 when Qoppa was evaluated as a classifier of death and 0.07 when assessed as a classifier of de novo metastasis development in patients without metastases at the time of sample collection. No classification role was observed for Qoppa or Stigma in the remaining possible scenarios. These findings are shown in Figure 3 and summarized in Table 5 for the cases in which Qoppa is discriminatory (AUC > 0.5; mortality in the overall cohort and development of de novo metastases among patients who were non-metastatic at the time of sample collection).

A more detailed analysis of Qoppa’s role as a classifier for death risk revealed that the AUC of the complete Qoppa is superior to the AUC values of the Qoppa components associated with global laboratory parameters (Ϙ_G_; AUC = 0.65) or biomarkers (Ϙ_B_; AUC = 0.69), separately. Furthermore, the role of the complete Qoppa as a classifier for mortality risk in the population who were exclusively non-metastatic at baseline increased to AUC = 0.98, whereas its classification role disappeared when considering the population who were exclusively metastatic at baseline (AUC = 0.44). A detailed analysis of Qoppa as a classifier for the risk of de novo metastasis in non-metastatic patients at baseline demonstrated a similar pattern. The AUC of the complete Qoppa was superior to the AUC values of the Qoppa components associated with global laboratory parameters (Ϙ_G_; AUC = 0.72) or biomarkers (Ϙ_B_; AUC = 0.77). Subpopulation-specific ROC curves are available in the Supplementary Material.

The optimal cutoff point for Qoppa regarding the mortality risk was 4.775 (sensitivity, 1.00; and specificity 0.65. 95% CI: 4.774–6.195) and 4.949 (sensitivity, 0.80; and specificity 0.84. 95% CI: 3.55–8.48) for the risk of de novo metastasis development. The study of de novo metastasis development in the non-metastatic population at baseline involved a small subpopulation of the overall population. For clinical implementation and analytical consistency within this exploratory pilot study, we adopted the cutoff derived from the broader population (Qoppa > 4.775) as the single operational threshold. Therefore, when applying a cutoff point of 4.775 to the de novo metastasis study, we obtained the same sensitivity and specificity as those for the optimally calculated cutoff point of 4.949.

**Figure 4:**
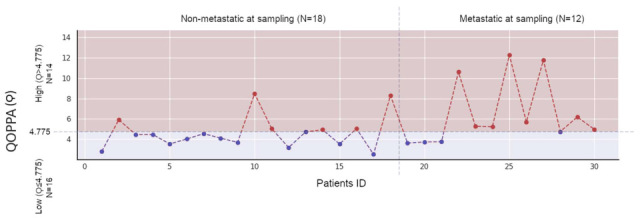
Qoppa synthetic cluster variable profile for all patients. The cutoff point obtained from the ROC curve study is indicated, defining the range of low Qoppa (Ϙ ≤ 4.775) and high Qoppa (Ϙ > 4.775). The non-metastatic population at sampling time is also identified in relation to the population with metastases at that time.

Based on these results, we stratified the population into low Qoppa (Ϙ ≤ 4.775, resulting in 16 patients), and high Qoppa (Ϙ > 4.775, resulting in the remaining 14 patients). These results are shown in Figure 4.

Analytical and clinical differences between the populations with high and low Qoppa were analyzed using the Mann–Whitney U test for variables that did not meet the normality criteria and with the Student’s t-test for those that did. Statistically significant analytical differences were observed in the distribution of the ANGPTL4, CATHEPSIN-D, FGF21, ICAM1, LAR, MPO, and NPM values. All these variables showed a non-normal distribution, and in every case, the median value was higher in the high-Qoppa group than in the low-Qoppa group. From a clinical variable standpoint, differences were observed between the high- and low-Qoppa groups in terms of the disease stage, tumor burden, and metastatic burden, at diagnosis, at sampling, and post-sampling, for all these parameters. In all cases, the high-Qoppa group demonstrated higher median values than the low-Qoppa group, with the exception of the weighted metastatic burden at diagnosis, where both groups exhibited a median of zero, although with different ranges (0–5 for the low-Qoppa group versus 0–12 for the high-Qoppa group). When analyzing the non-metastatic subgroup at sampling, significant differences between high- and low-Qoppa patients were observed in the stage, tumor burden, post-sampling metastatic burden, and de novo metastatic development. In the metastatic subgroup at sampling, differences were restricted to the stage and metastatic burden at diagnosis. None of the clinical variables demonstrated a normal distribution. The analytical and clinical differences analyses are available in the Supplementary Material.

**Figure 5:**
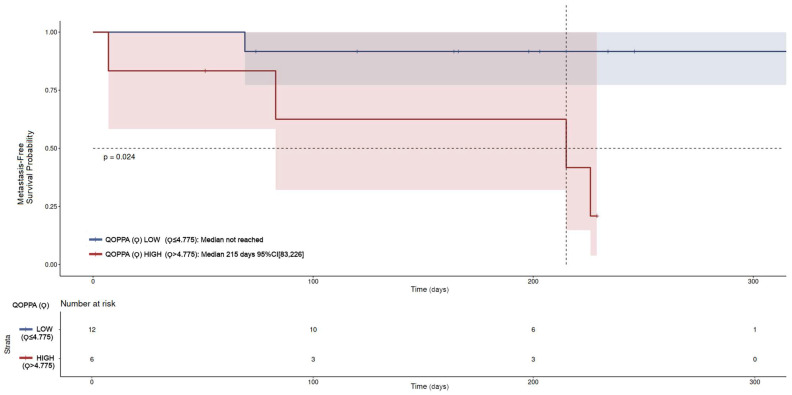
Kaplan–Meier metastasis-free survival analysis between the non-metastatic population at sampling with high Qoppa (Ϙ > 4.775, six patients) and low Qoppa levels (Ϙ ≤ 4.775, twelve patients). Statistically significant differences were observed with *p*-value 0.024. The median metastasis-free survival for the group of high Qoppa levels was 215 days with 95% CI: 83–226. The median metastasis-free survival for the group of low Qoppa was not reached. The corresponding confidence intervals are depicted for each curve as shaded areas.

The survival analysis for metastasis-free survival (MFS) using the Kaplan–Meier methodology in the non-metastatic subpopulation at sampling (N = 18), stratified by Qoppa level (high N = 6, low N = 12), demonstrated statistically significant differences. The median MFS was 215 days in the high-Qoppa group versus not reached (>300 days) in the low-Qoppa group (*p* = 0.024, Figure 5). However, Cox proportional hazards analysis did not reach statistical significance (HR = 8.4, 95% CI: 0.93–76, *p* = 0.058). The proportional hazards assumption was not violated (Schoenfeld test *p* = 0.49).

**Figure 6:**
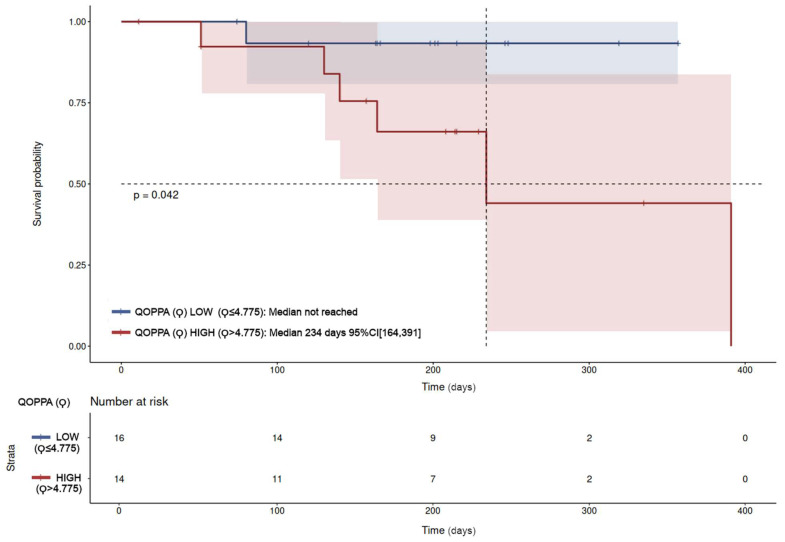
Kaplan–Meier overall survival analysis between the population with high Qoppa (Ϙ > 4.775, 14 patients) and low Qoppa levels (Ϙ ≤ 4.775, 16 patients). Statistically significant differences were observed with *p*-value 0.042. The median survival for the group of high Qoppa levels was 234 days with 95% CI: 164–391. The median survival for the group of low Qoppa was not reached. The corresponding confidence intervals are depicted for each curve as shaded areas.

The Kaplan–Meier analysis of overall survival was performed in the total population stratified by Qoppa value (high vs. low), demonstrating statistically significant differences. The median overall survival was 234 days in the high-Qoppa group versus not reached in the low-Qoppa group (*p* = 0.042, Figure 6). However, Cox proportional hazards analysis did not achieve statistical significance (HR = 6.9; 95% CI: 0.8–0.59; *p* = 0.079). The proportional hazards assumption was assessed using Schoenfeld residuals; no violations were detected (*p* = 0.29). A detailed analysis of the non-metastatic and metastatic subpopulations at sampling revealed that statistically significant differences in the mortality risk disappeared in the Kaplan–Meier analysis for the metastatic-at-sampling population. Conversely, these differences were confirmed in the non-metastatic-at-sampling population, showing an overall survival median difference of 391 days for the high-Qoppa population versus not reached in the low-Qoppa group (*p* = 0.034), with complete separation observed in the Cox proportional hazards analysis. These subpopulation analyses are available in the Supplementary Material.

## 4. Discussion

This prospective pilot study indicates that Qoppa, a synthetic parameter that integrates organ-derived response biomarkers with global laboratory parameters, exhibits satisfactory discriminatory performance (AUC = 0.78) in predicting de novo metastasis and mortality among patients with solid tumors. This is supported by the statistically significant differences observed in the Kaplan–Meier analysis. Qoppa is conceptually aligned with emerging pan-cancer biomarkers integrating organ-specific responses to estimate the metastatic risk. Most available multi-cancer approaches based on plasma proteomics [76], exosomal RNA signatures [77], and cfDNA methylation [78,79] panels have demonstrated a clinically relevant stratification across tumor types, supporting the rationale for such scalable assays and integrative models, as illustrated by recent work in other tumor settings [80,81,82].

The plasmatic biomarker panel integrated into Qoppa was intentionally designed to cover complementary dimensions of the premetastatic niche and systemic tumor interactions. ANGPTL4, HGF, and ICAM1 serve as pivotal mediators of endothelial activation and cell adhesion, thereby facilitating organ-specific extravasation of circulating tumor cells [31,32,39,40,41]. Cathepsin D and MPO capture proteolytic remodeling and neutrophil-driven inflammation, both associated with increased invasiveness and metastatic seeding [33,34,52,53]. FGF21, GDF15, and leptin integrate tumor-induced metabolic stress and adipose–hepatic signaling, processes that affect immune competence and the metabolic state of target organs [35,36,37,38,50,51]. Additionally, IL-6, IL-10, and IL-18 were selected as cytokines with dual pro-tumor and immunoregulatory functions, collectively assessing chronic systemic inflammation, and immune escape [42,43,44,45,46,47,48,83]. Beyond their mechanistic significance, these biomarkers were selected based on prior evidence of dynamic variation with tumor burden, partial enrichment in liver, adipose, or endothelial tissues that enable organ-related inference, and their feasibility for robust multiplex quantification in clinical laboratories. Collectively, Qoppa relies on standard blood sampling, available biochemical parameters, and a biomarker panel, a multiplex platform that can be performed by an external laboratory. Whereas other studies assessed multiplex immunofluorescence [84], this study is the first to systematically integrate multiorgan-derived response biomarkers with global laboratory parameters for metastatic risk stratification across multiple tumor types.

The Qoppa parameter, comprising 11 biomarkers responsive to tumor-secreted soluble factors and 16 global laboratory parameters, was internally validated, showing significant variation according to the prognostic risk. Furthermore, the differences between high and low Qoppa values reflected marked divergence in certain biomarkers responsive to tumor-secreted soluble factors. Additionally, the differences between high and low Qoppa were correlated with clinical differences in disease stage, tumor burden, and metastatic burden at diagnosis, sampling, and post-sampling. Thus, these findings reinforce the concept that the variables comprising the synthetic Qoppa parameter capture the liquid biopsy expression of disease trajectory characteristics and the oncologic process, enabling repeated non-invasive monitoring of high-risk patients [85].

The results presented herein should be considered hypothesis-generating for subsequent larger studies concerning the clinical utility of Qoppa. Adopting Qoppa into real-world practice would require, first, a clinical validation in higher cohorts, a cost study, as well as standardization of the analytical procedures (sample handling, calibration, quality control) [86]. Indeed, there is a risk of model overfitting, which was partially mitigated by internal validation using bootstrap resampling, but this cannot substitute for external validation. Consistent with this, the Cox regression significance was borderline (*p* = 0.058), which likely reflects insufficient power rather than the absence of association. Importantly, this approach contributes to establishing a novel ultra-early detection paradigm, aligning with the most innovative and current trends in oncology, wherein tumor disease is identified prior to its visibility on imaging studies [87].

Qoppa exhibited limited discriminatory capacity in patients with metastatic disease at the time of analysis. Given that Qoppa integrates biomarkers of the response to soluble tumor factors and global analytical parameters, this finding suggests that while this approach can detect metastatic onset, it is inadequate for identifying variations in metastatic burden once the metastatic process is established. This predictive limitation in the metastatic context may be attributable to the higher biological complexity inherent to advanced disease compared with at-risk populations with localized disease [88]. In established metastases, multiple progression pathways and molecular mechanisms operate concurrently, generating substantial “biological noise” that obscures individual biomarker prognostic signals [89]. Altered biomarker values in this population likely reflect the manifestations of already-present advanced disease rather than capacity to predict subsequent progression. This indicates that this strategy should be complemented with additional biomarkers or monitoring approaches in the metastatic population to track the disease evolution [90]. Nevertheless, given the substantial prognostic impact of metastatic onset, Qoppa remains of considerable interest and potential clinical utility.

This pilot study has several limitations, primarily related to the limited statistical power (small sample size). First, the cohort size was modest (*n* = 30), and it was highly heterogeneous in terms of primary tumor type, stage, prior treatments, and metastatic burden. Although this heterogeneity is conceptually aligned with the pan-tumor and host-centered nature of Qoppa, it likely increased noise and diluted potential associations within specific subgroups. Importantly, such heterogeneity also limits the definition of a single universal cutoff, as clinical thresholds may vary across tumor types and clinical conditions. Second, given the small sample size compared to the number of integrative variables, there is a risk of model overfitting, which was partially mitigated by internal validation using bootstrap resampling, but this cannot substitute for external validation. As a consequence, the effect estimates, proposed cutoffs, and the apparent prognostic separation between Qoppa strata should be interpreted with caution and considered exploratory. Third, we did not pre-specify tumor-site-specific models; so, the clinical applicability of Qoppa remains uncertain. Before routine use, its prognostic performance, reproducibility and added value beyond standard clinicopathological variables should be confirmed in larger multicenter cohorts, ideally with prespecified tumor type and clinical scenarios. Future studies should also explore whether simplified context-specific versions of the biomarker panel can retain most of the predictive information while improving the feasibility and cost-effectiveness prior to clinical implementation.

## 5. Conclusions

This prospective pilot study suggests that the clinical measurement of response biomarkers to tumor-secreted soluble factors is feasible in peripheral blood from oncologic patients. Integration of these plasma biomarkers related to endothelial activation, inflammation, and metabolism with global laboratory parameters into the synthetic variable Qoppa was consistently associated with adverse risk and de novo metastasis in this small hypothesis-generating cohort, supporting the methodological feasibility of this approach. Although the present data are insufficient to justify immediate clinical implementation, they provide proof of concept that such integrative plasmatic panels can capture clinically relevant information in standard clinicopathological settings and warrant prospective validation and refinement in larger tumor-type-stratified and multicenter cohorts.

## Figures and Tables

**Table 1:** Biomarkers of response to tumor-secreted soluble factors. The aliases for response biomarkers are written in uppercase in this study.

Biomarker	Alias	Description
Angiopoietin-like 4	ANGPTL4	Expressed in hepatic and adipose tissues. It exhibits heterogeneous pro-angiogenic roles in metastatic disease progression [31,32].
Cathepsin D	CATD	A lysosomal protease secreted by tumor cells. It functions as a promoter of cancer cell replication, invasive phenotype, and metastatic dissemination [33,34].
Fibroblast Growth Factor 21	FGF21	A hepatokine regulated by nutritional status and hypoxia also secreted by tumor microenvironment fibroblasts. It acts as a pivotal immune suppressor leading to CD8+ T cells exhaustion [35,36].
Growth/Differentiation Factor 15	GDF15	A member of the transforming growth factor-β superfamily. It is upregulated in systemic inflammation and metabolic response to tumor burden [37,38].
Hepatocyte Growth Factor	HGF	A multifunctional growth factor. It exhibits pro-angiogenic, pro-invasive, and pro-metastatic functions through c-MET signaling pathway activation [39,40].
Intercellular Adhesion Molecule 1	ICAM1	An adhesion molecule constitutively expressed on endothelial cells and leukocytes. It is dysregulated in tumor-associated inflammation and participates in premetastatic niche formation [41,42].
Interleukin 6	IL6	Pro-inflammatory interleukin that activates STAT3 via JAK signaling in both tumor cells and stromal cells. It facilitates epithelial-mesenchymal transition and suppresses cytotoxic T lymphocyte-mediated anti-tumor immunity [43].
Interleukin 10	IL10	Anti-inflammatory interleukin with paradoxical pro-metastatic activity in malignancy. It promotes immune escape through T regulatory cell amplification, PD-L1 upregulation on monocytes, and suppression of CD8+ T cell infiltration and cytotoxic anti-tumor immunity [44,45].
Interleukin 18	IL18	Pro-inflammatory interleukin with paradoxical stage- and tissue-dependent functionality. It may suppress the primary tumor, however, may also enhance the invasion ability in metastatic stage [45,46,47,48,49].
Leptin	LEP	An adipokine with pleiotropic roles in metabolic and immune regulation. It is dysregulated by metabolic augmentation secondary to tumor-derived systemic effects [50,51].
Myeloperoxidase	MPO	An enzyme constitutively expressed in neutrophil granulocytes. It serves as a circulating marker of neutrophilic infiltration and inflammatory cell invasion associated with tumor progression [52,53].

**Table 2:** Global analytical parameters derived from routine laboratory assessments. The aliases for global laboratory parameters are written in lowercase in this study.

Parameter	Alias	Description
Albumin	alb	Serum albumin concentration in peripheral venous blood (g/dL) [23,54].
Aspartate aminotransferase—platelet count ratio	apri	Aspartate aminotransferase–platelet ratio (apri = AST/ULN of AST/absolute platelet count [10^9^/L] × 100) [21,22].
Aspartate aminotransferase–neutrophil ratio	anri	Aspartate aminotransferase–neutrophil ratio (anri = AST/ULN/absolute neutrophil count [10^9^/L]) [21,22,55,56].
Hemoglobin–albumin–lymphocyte–platelet	halp	Hemoglobin–albumin–lymphocyte–platelet index (halp = 100 × Hemoglobin [g/dL] × Albumin [g/dL]/Absolute peripheral blood lymphocytes × Absolute peripheral blood platelets) [57,58,59].
Hemoglobin	hb	Serum hemoglobin concentration in peripheral venous blood (g/dL) [60,61].
Lactate dehydrogenase–albumin ratio	lar	Lactate dehydrogenase–albumin ratio (lar = Lactate dehydrogenase/Albumin) [62].
Lymphocyte–monocyte ratio	lmr	Lymphocyte–monocyte ratio
		(lmr = Absolute peripheral blood lymphocytes/Absolute peripheral blood monocytes) [21,22,63].
Leukocyte–lymphocyte ratio	llr	Leukocyte–lymphocyte ratio (llr = Absolute peripheral blood leukocytes/Absolute peripheral blood lymphocytes) [21,22].
Neutrophil–lymphocyte ratio	nlr	Neutrophil–lymphocyte ratio (nlr = Absolute peripheral blood neutrophils/Absolute peripheral blood lymphocytes) [23,58,60,64,65,66,67].
Naples prognostic score	nps	Naples prognostic score (nps = Σ nps_i_ for i between 1 and 4, where nps_1_ = 1 if alb ≥ 4 mg/dL, nps_2_ = 1 if total cholesterol ≤ 180 mg/dL, nps_3_ = 1 if nlr > 2.96, and nps_4_ = 1 if lmr ≥ 4.44) [63].
C-reactive protein	pcr	C-reactive protein concentration in peripheral venous blood [68].
Neutrophil–platelet–lymphocyte–hemoglobin ratio	nplhb	Neutrophil–platelet–lymphocyte–hemoglobin ratio (nplhb = Absolute peripheral blood neutrophils × Absolute peripheral blood platelets/Absolute peripheral blood lymphocytes × Hemoglobin) [69].
Novel prognostic model	npm	Novel prognostic model (npm = Σ npm_i_ for I between 1 and 2, where npm_1_ = 1 if nplhb ≥ 5.667 and npm_2_ = 1 if absolute peripheral blood monocytes ≥ 0.5051/mL) [69].
Platelet–lymphocyte ratio	plr	Platelet–lymphocyte ratio (plr = Absolute peripheral blood platelets/Absolute peripheral blood lymphocytes) [23,58,64,65,67].
Prognostic nutritional index	pni	Prognostic nutritional index (pni = 5 × Absolute lymphocytes [10^9^/L] − 10 × Albumin [g/dL]) [59,65,67,70].
Serum iron	si	Serum iron concentration [71].
Systemic immune inflammation index	sii	Systemic immune inflammation index (sii = Absolute peripheral blood neutrophils [/L] × Absolute peripheral blood platelets [/L]/Absolute peripheral blood lymphocytes [/L]) [21,22].
Systemic inflammation response index	siri	Systemic inflammation response index (siri = Absolute peripheral blood neutrophils [/L] × Absolute peripheral blood monocytes [/L]/Absolute peripheral blood lymphocytes [/L]) [72].
Combined platelet–NLR score	copnlr	Combined platelet–nlr score (cop-nlr = Σ copnr_i_ for i between 1 and 2, where copnr_1_ = 1 if nlr > 3 and copnr_2_ = 1 if absolute platelets > 300 × 10^9^/L) [21,22].
Vitamin B12	vitb12	Serum vitamin B12 concentration in peripheral blood (pg/mL) [73].

**Table 3:** Demographic clinical characteristics of the study population (*n* = 30).

Characteristic	Value
Recruited population (N)	30
Sex (Female:Male)	17:13
Age at diagnosis, years (median [range])	64.44 [32.31–79.52]
Histology	
Adenocarcinoma	8
Endometrioid adenocarcinoma	1
Serous adenocarcinoma	1
Clear cell carcinoma	1
Invasive ductal carcinoma	5
Squamous cell carcinoma	1
Invasive lobular carcinoma	2
Neuroendocrine carcinoma	4
High grade serous carcinoma	1
Urothelial carcinoma	4
Cholangiocarcinoma	1
Liposarcoma	1
Origin	
Colon	3
Cervix	1
Endometrium	2
Esophagus	1
Breast	7
Pancreas	2
Prostate	1
Lung	4
Retroperitoneal	1
Kidney	2
Bladder	3
Gallbladder	1
Distal biliary tract	1
Ovary	1
Stage at diagnosis (median)	2.5
Tumor burden at diagnosis (median [range])	1 [1–13]
Metastasis at diagnosis (N (%))	7 (23%)
Metastatic burden at diagnosis (median [range])	0 [0–12]
Stage at sample collection (median)	3.5
Tumor burden at sample collection (median [range])	2 [0–14]
Metastasis at sample collection (N (%))	12 (40%)
Metastatic burden at sample collection (median [range])	0 [0–12]
Stage at post-sampling (median)	4
Tumor burden at post-sampling (median [range])	2 [0–14]
Metastasis at post-sampling (N (%))	17 (57%)
Metastatic burden at post-sampling (median [range])	1 [0–12]
Stage at end of follow-up (median)	4
Tumor burden at end of follow-up (median [range])	2 [0–22]
Metastasis at end of follow-up (N (%))	17 (57%)
Metastatic burden at end of follow-up (median [range])	1 [0–20]
Death (N (%))	7 (23%)

**Table 4:** Composition of the synthetic study variables Stigma (Ϛ) and Qoppa (Ϙ), as well as the contribution of the global laboratory parameters and the response biomarkers to Qoppa (Ϙ_G_ and Ϙ_B_, respectively).

Synthetic Study Variable	Symbol	Number of Parameters	Parameters
Stigma	Ϛ	5	nps + lmr + alb + hb + pni
Qoppa	Ϙ (Ϙ = Ϙ_G_ + Ϙ_B_)	26	copnlr + plr + nplhb + sii + siri + nlr + llr + anri + ICAM1 + IL6 + MPO + HGF + CATHEPSIN-D + pcr + ANGPTL4 + apri + IL10 + IL18 + FGF21 + GDF15 + npm + lar + LEPTIN + si + halp + vitb12
Qoppa global laboratory parameters’ contribution	Ϙ_G_	15	copnlr + plr + nplhb + sii + siri + nlr + llr + anri + pcr + apri + npm + lar + si + halp + vitb12
Qoppa response biomarkers’ contribution	Ϙ_B_	11	ICAM1 + IL6 + MPO + HGF + CATHEPSIN-D + ANGPTL4 + IL10 + IL18 + FGF21 + GDF15 + LEPTIN

**Table 5:** Main characteristics and performance of the ROC operator curves for the case studies described in Figure 3. Optimism correction with bootstrapping using 1000 bootstraps is applied, showing the corrected area under the curve with its 95% confidence interval. The *p*-value for comparing the ROC curve against no discriminatory ability (AUC = 0.5) using the Mann–Whitney U test is also shown. The sensitivity and specificity at the cutoff point of 4.775 is also presented.

Case Study	Sample Size (N)	Original AUC	Optimism Corrected AUC	95% Confidence Interval	*p*-Value	Sensitivity	Specificity
Qoppa for risk of death (Figure 3a)	30	0.78	0.77	0.60–0.92	0.03	1	0.65
Qoppa for risk of development of metastasis de novo in patients with no metastasis at sample collection (Figure 3c)	18	0.78	0.77	0.48–1.0	0.07	0.8	0.84

## Data Availability

The min–max scaled raw data are available as Supplementary Material.

## Supplementary Materials

The following supporting information can be downloaded at https://www.mdpi.com/article/10.3390/jcm15020846/s1, RAW_DATA_MINMAXSCALED; Table S1: Descriptive values of the 11 biomarkers measured through Luminex; Table S2: Main characteristics and performance of the ROC operator curves for the case studies described as supplementary material with optimism correction bootstrapping; Table S3: Main characteristics of the study population regarding follow-up time, received treatments before sample collection, and presence of metastases at the time of sample collection; Table S4: Summary of metastatic sites and number of metastases per location and calculation of weighted metastatic burden and overall tumor burden. Figure S1: Profile of synthetic variable values of clusters Stigma (Ϛ) and Qoppa (Ϙ); Figure S2: ROC curve analysis study of the role of the component of Qoppa from global analytical parameters (Ϙ_G_, panel a) and from response biomarkers (Ϙ_B_, panel b) as a classifier of the risk of death; Figure S3: ROC curve analysis study of the role of Qoppa as a classifier of the risk of death for patients with no metastasis at the time of sample collection (panel a) and patients with metastasis at the time of sample collection (panel b); Figure S4: ROC curve analysis study of the role of the component of Qoppa from global analytical parameters (ϘG, panel a) and from response biomarkers (ϘB, panel b) as a classifier of the risk of development of metastasis de novo in patients with no metastasis at the time of sample collection; Figure S5: Study of analytical differences using the Mann–Whitney U test for variables that did not meet the normality criteria and the Student’s t test for those that did; Figure S6: Study of clinical differences using the Mann–Whitney U test for variables that did not meet the normality criteria and the Student’s t test for those that did; Figure S7: Kaplan–Meier survival analysis between the patients with high and low Qoppa levels for the population with no metastasis at the time of sample collection; Figure S8: Kaplan–Meier survival analysis between the patients with high and low Qoppa levels for the population with metastasis at the time of sample collection.
