# Supplementary material for: Qoppa as a New Pan-Tumor Synthetic Parameter Derived from Tumor-Associated Biomarkers for Identifying Oncology Patients at High Risk of Metastasis: A Prospective Pilot Study
Source: J Clin Med. 2026 Jan 20;15(2):846. doi: 10.3390/jcm15020846 (PMC12841959; doi:10.3390/jcm15020846)
Supplement: Supplementary file 1 [file jcm-15-00846-s001.zip › DIAZSANTOSetal_Supplementary_FigureS1.docx]

Article

Qoppa as a New Pan-Tumor Synthetic Parameter Derived from Tumor-Associated Biomarkers for Identifying Oncology
Patients at High Risk of Metastasis: A Prospective Pilot Study

Javier Diaz-Santos ^1,2,^*, Alba Rodriguez-Valle ^1,2^, Beatriz Berrocal-Gavilan ^1,2^, Olivia Urquizar-Rodriguez ^1,2^
and Silvia Montoro-Garcia ^3^

|  |
| --- |


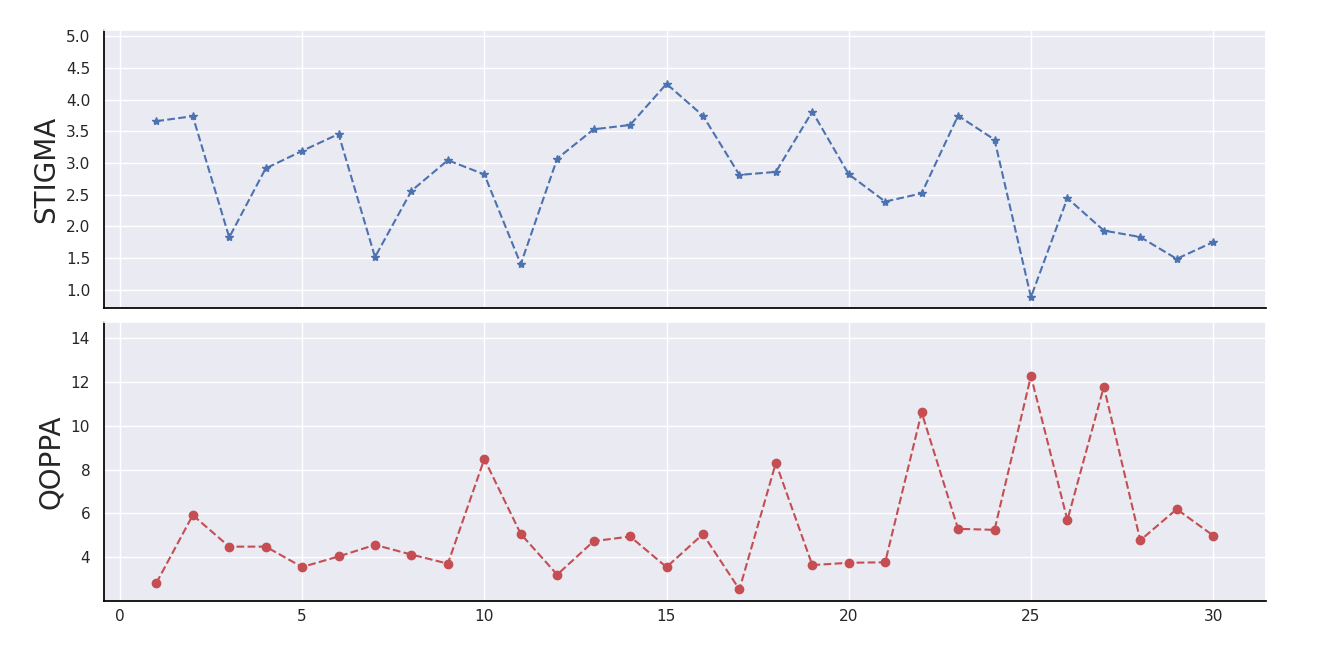


**Figure S1****.** Profile of synthetic variable values of clusters Stigma (Ϛ) and Qoppa (Ϙ)
