# Supplementary material for: Qoppa as a New Pan-Tumor Synthetic Parameter Derived from Tumor-Associated Biomarkers for Identifying Oncology Patients at High Risk of Metastasis: A Prospective Pilot Study
Source: J Clin Med. 2026 Jan 20;15(2):846. doi: 10.3390/jcm15020846 (PMC12841959; doi:10.3390/jcm15020846)
Supplement: Supplementary file 1 [file jcm-15-00846-s001.zip › DIAZSANTOSetal_Supplementary_FigureS2.docx]

Article

Qoppa as a New Pan-Tumor Synthetic Parameter Derived from Tumor-Associated Biomarkers for Identifying Oncology
Patients at High Risk of Metastasis: A Prospective Pilot Study

Javier Diaz-Santos ^1,2,^*, Alba Rodriguez-Valle ^1,2^, Beatriz Berrocal-Gavilan ^1,2^, Olivia Urquizar-Rodriguez ^1,2^
and Silvia Montoro-Garcia ^3^

| 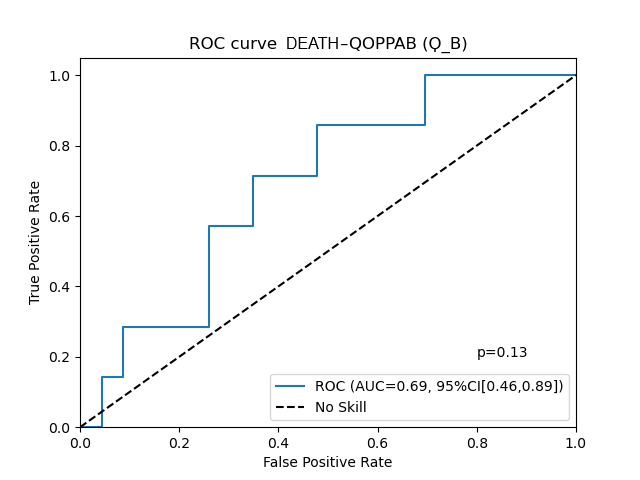(**b**) | 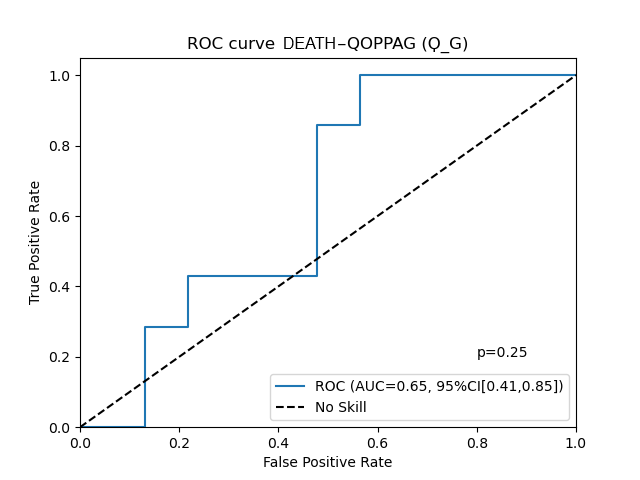(**a**) |
| --- | --- |

**Figure S****2.** ROC curve analysis study of the role of the component of Qoppa from global analytical parameters (Ϙ_G_, panel a) and from response biomarkers (Ϙ_B_, panel b) as a classifier of the risk of death. For each case, the 95% confidence interval and p-value for comparing the ROC curve against no discriminatory ability (AUC=0.5) using the Mann-Whitney U test are also shown.
