# Supplementary material for: Qoppa as a New Pan-Tumor Synthetic Parameter Derived from Tumor-Associated Biomarkers for Identifying Oncology Patients at High Risk of Metastasis: A Prospective Pilot Study
Source: J Clin Med. 2026 Jan 20;15(2):846. doi: 10.3390/jcm15020846 (PMC12841959; doi:10.3390/jcm15020846)
Supplement: Supplementary file 1 [file jcm-15-00846-s001.zip › DIAZSANTOSetal_Supplementary_FigureS6.docx]

Article

Qoppa as a New Pan-Tumor Synthetic Parameter Derived from Tumor-Associated Biomarkers for Identifying Oncology
Patients at High Risk of Metastasis: A Prospective Pilot Study

Javier Diaz-Santos ^1,2,^*, Alba Rodriguez-Valle ^1,2^, Beatriz Berrocal-Gavilan ^1,2^, Olivia Urquizar-Rodriguez ^1,2^
and Silvia Montoro-Garcia ^3^


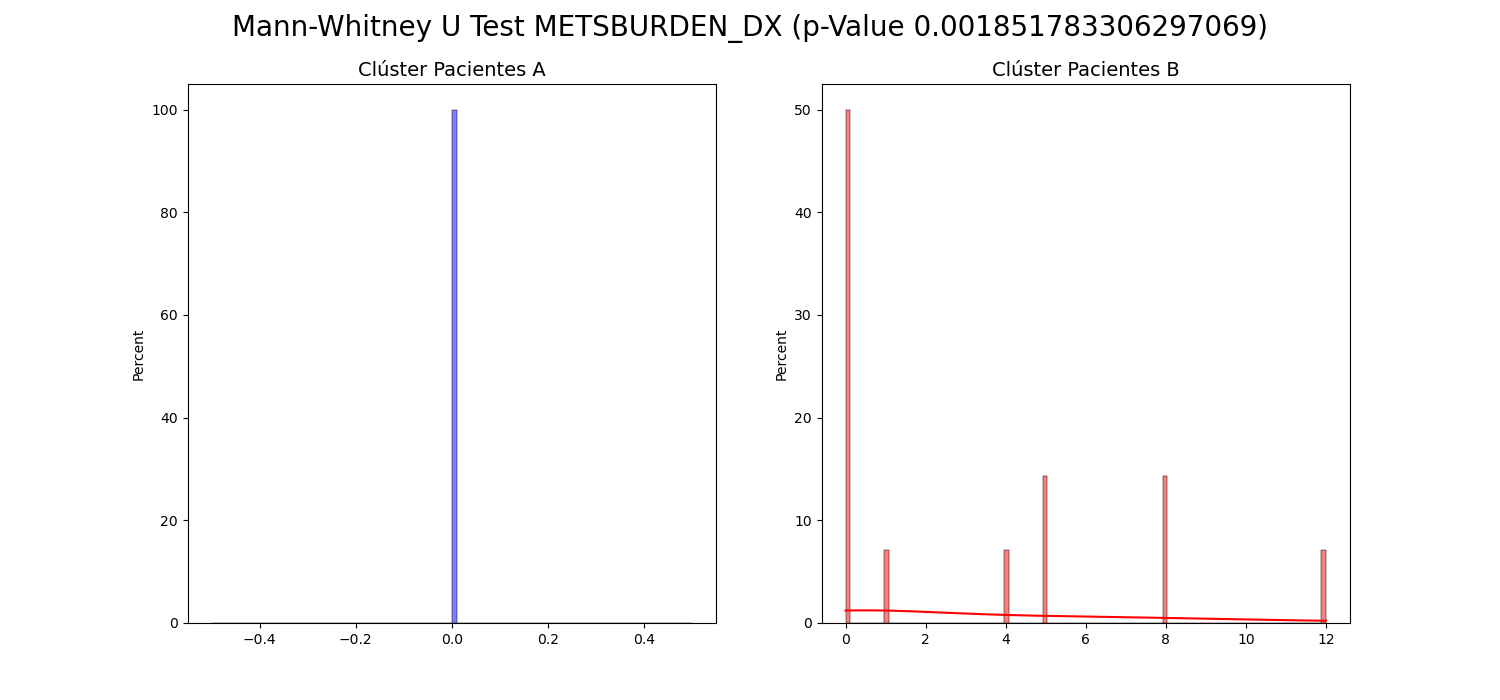

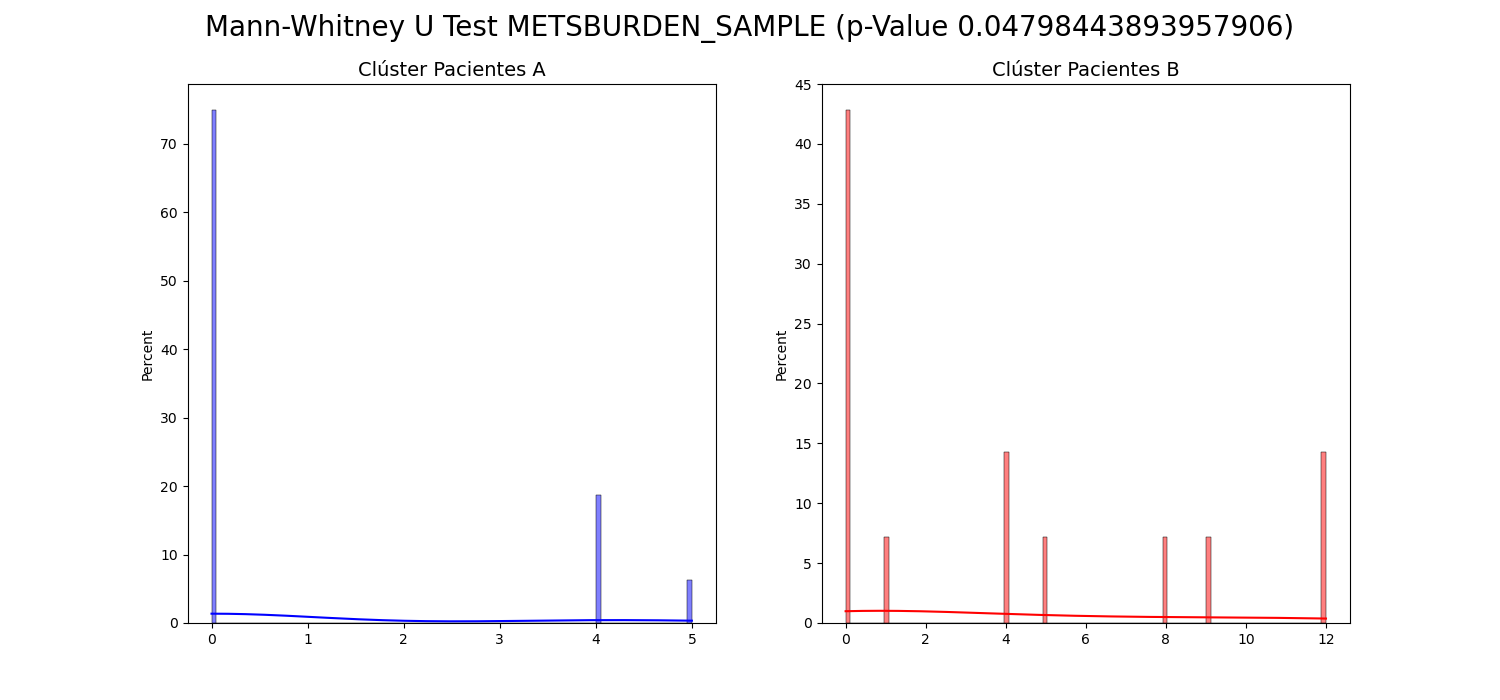

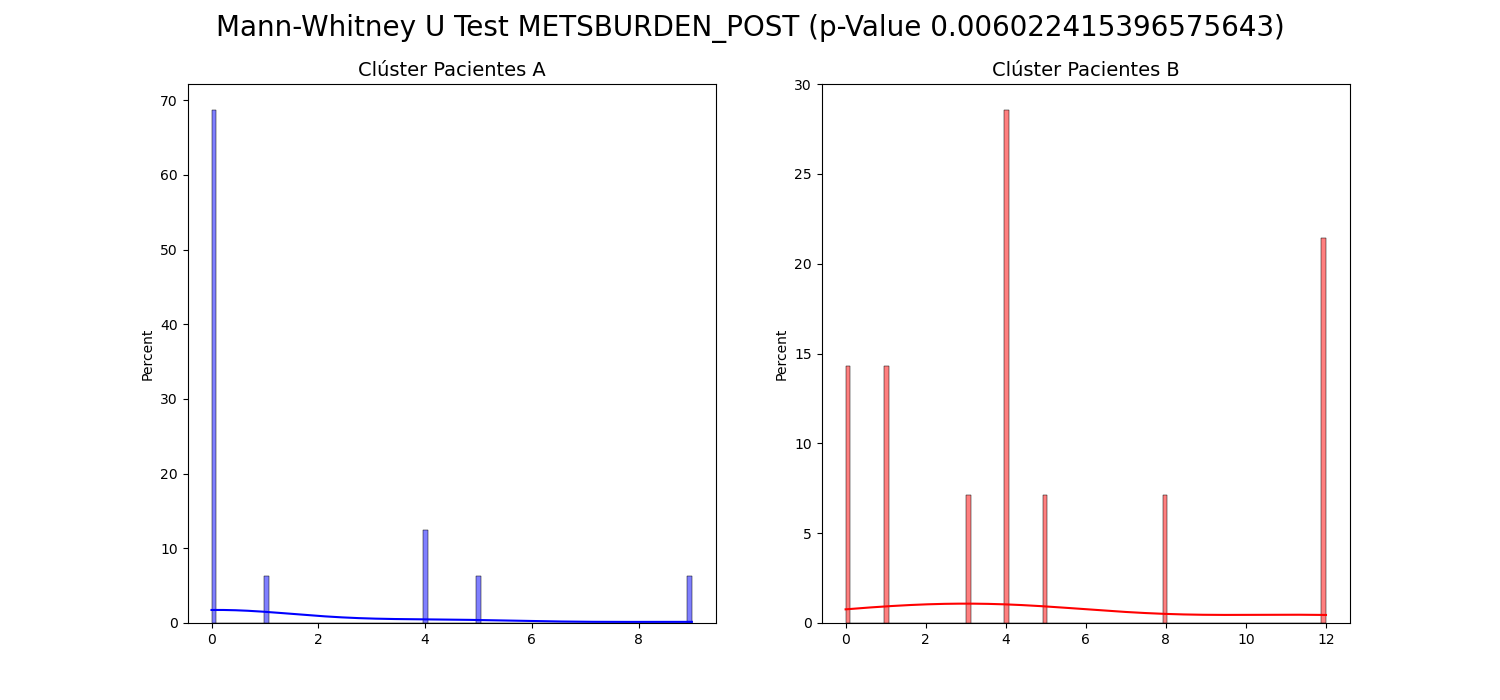

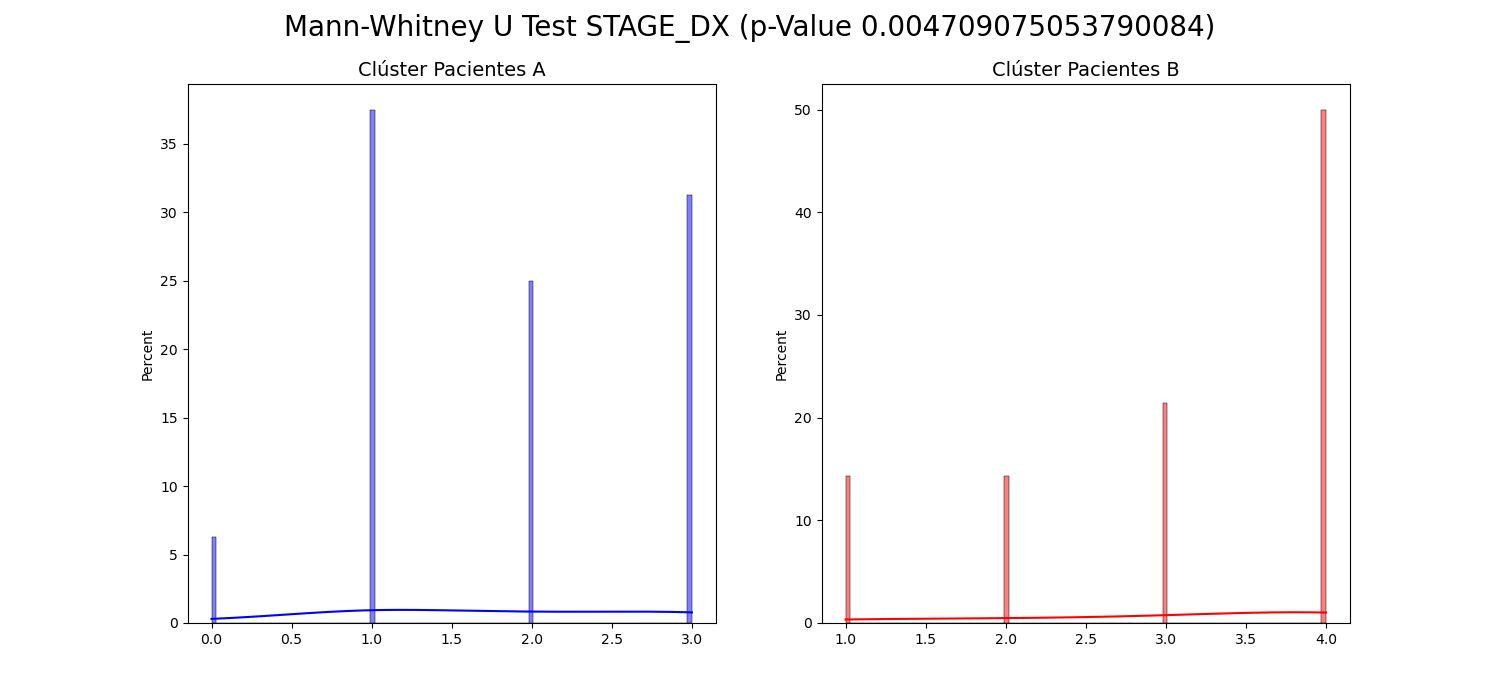

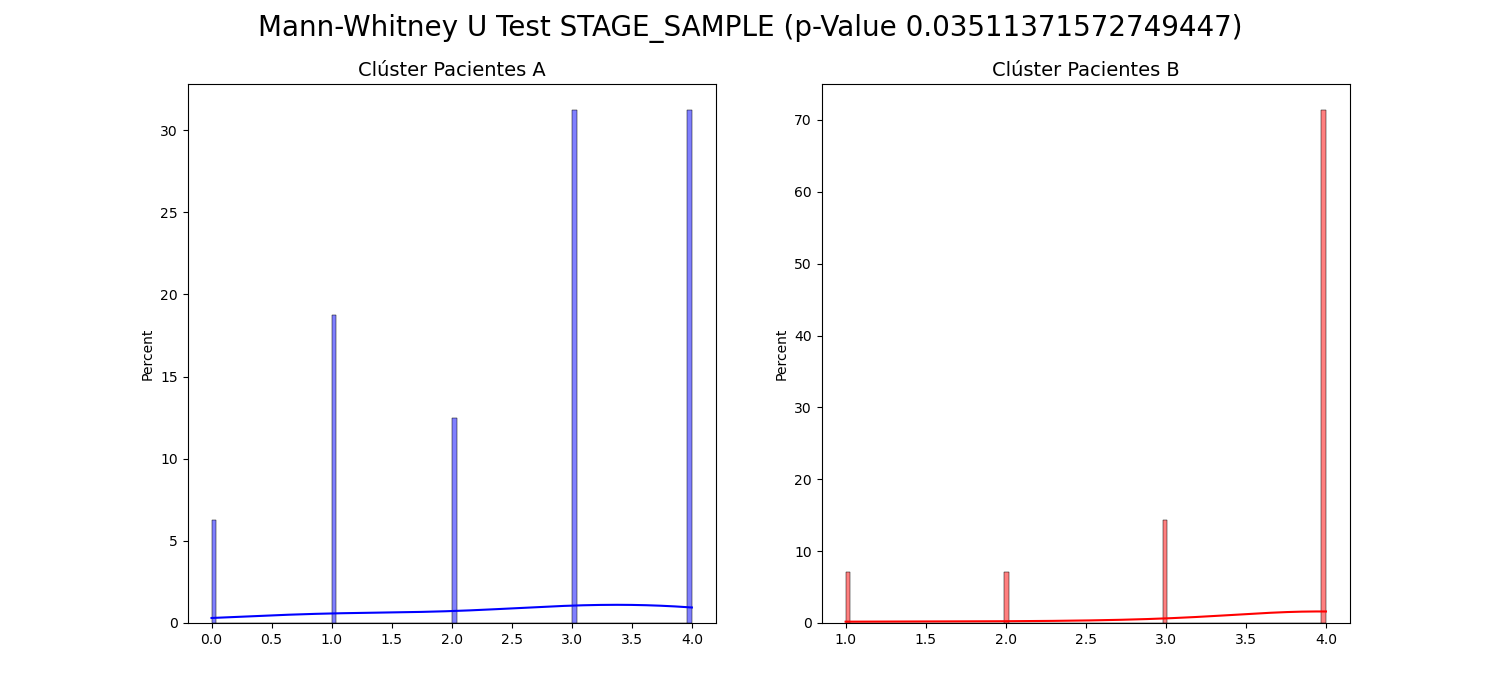

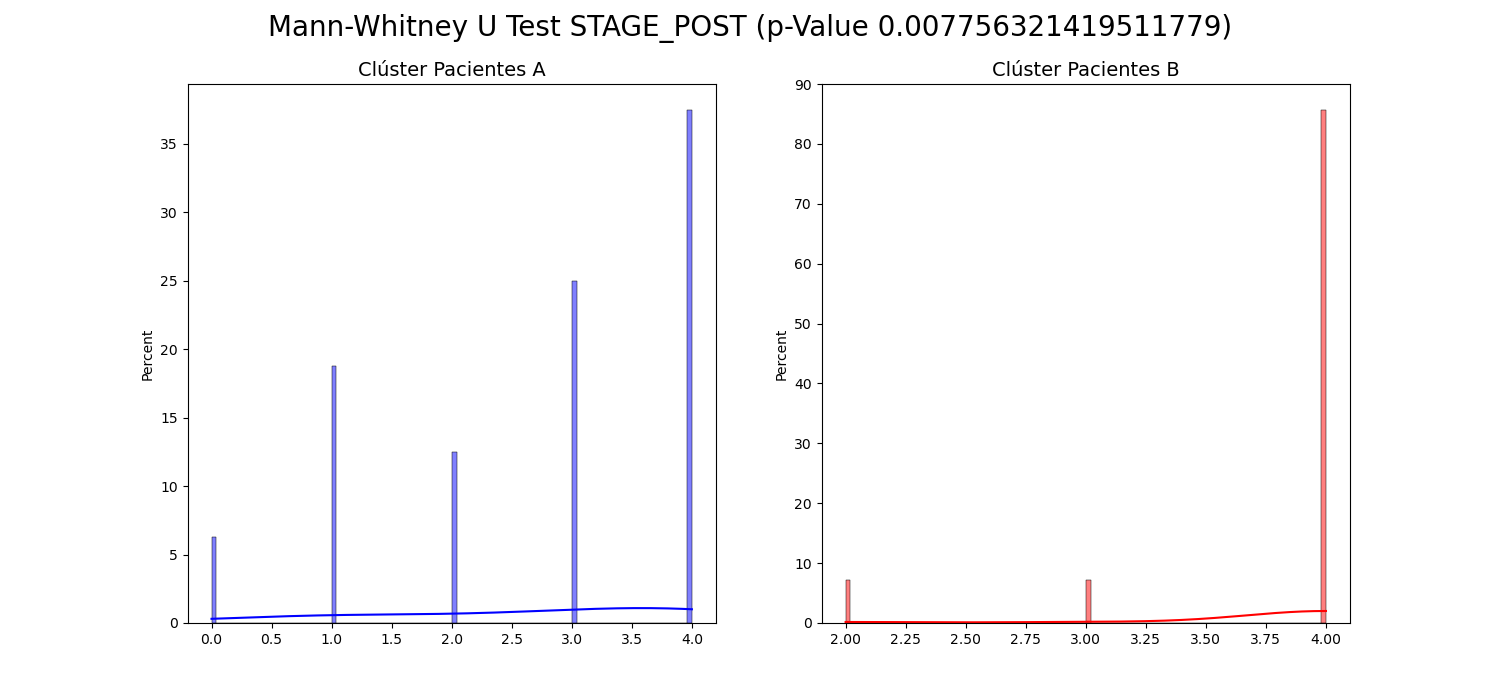

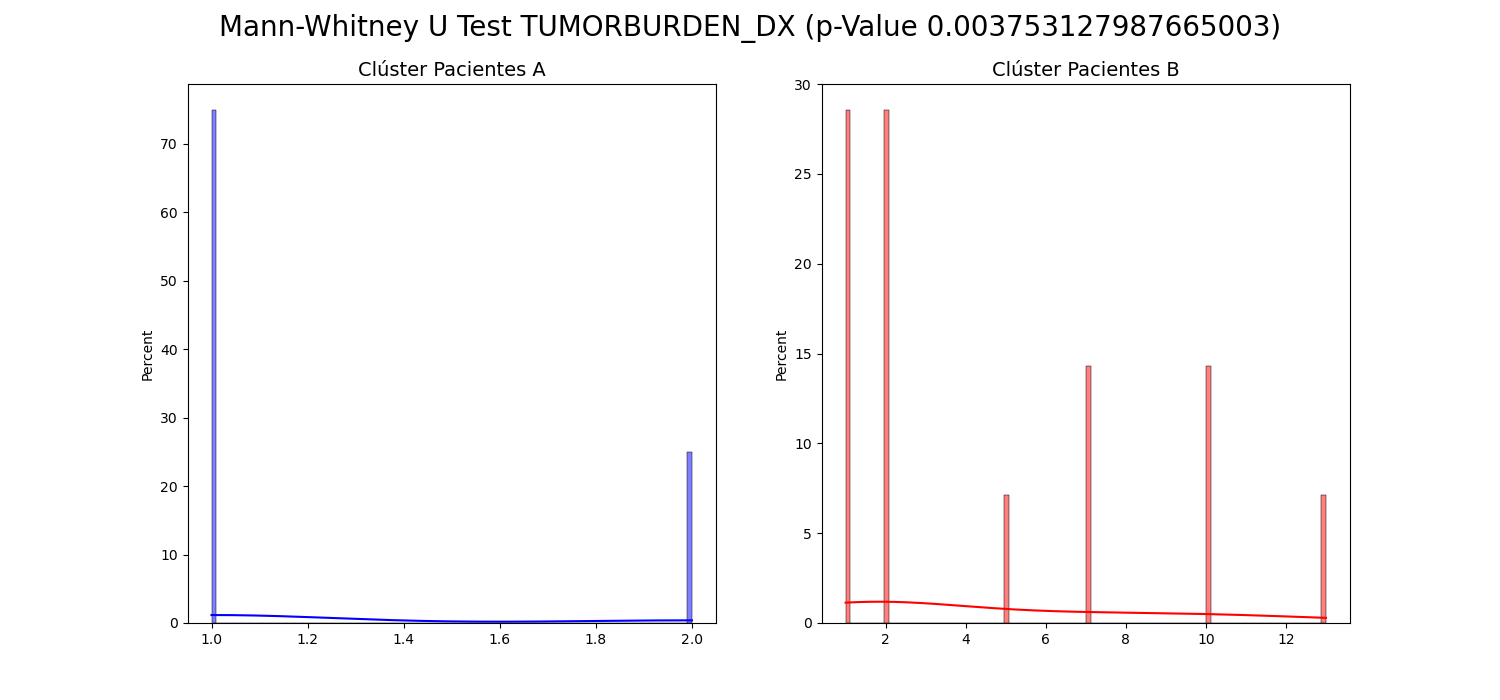

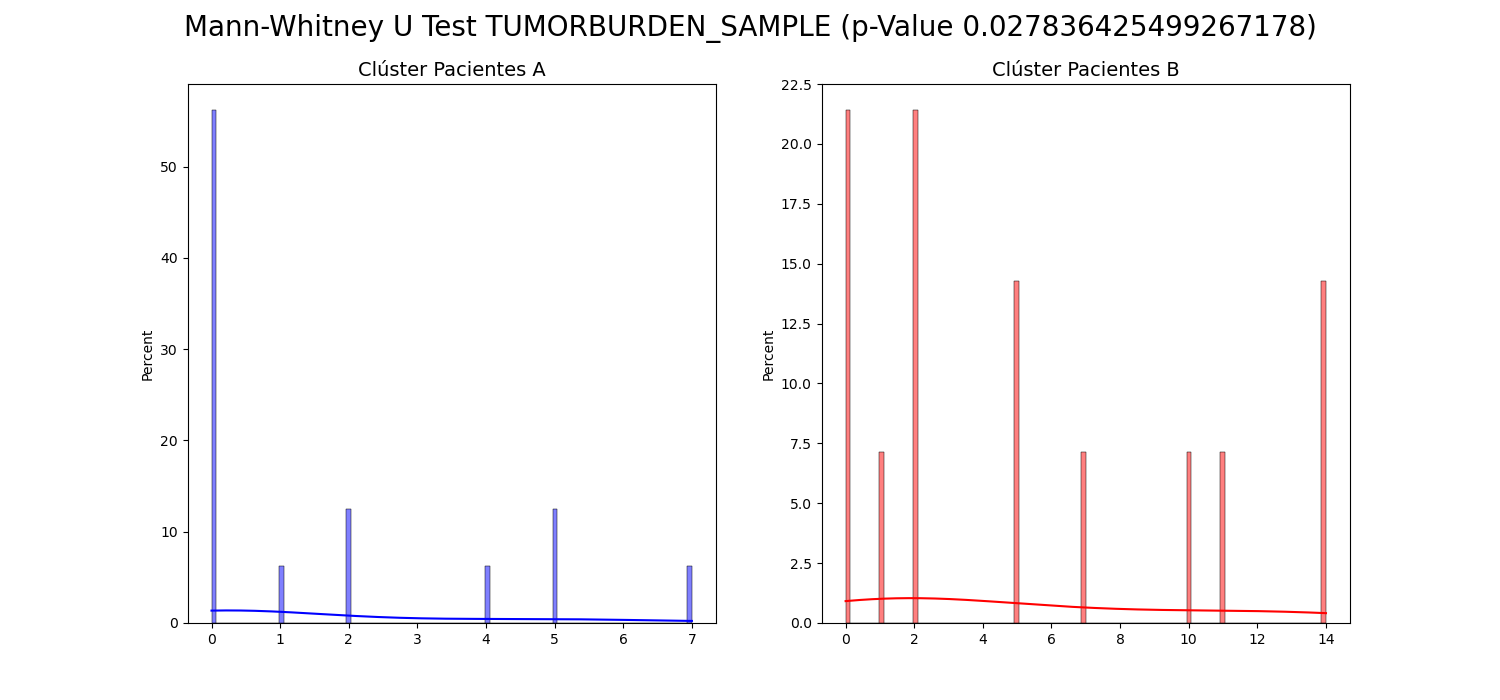

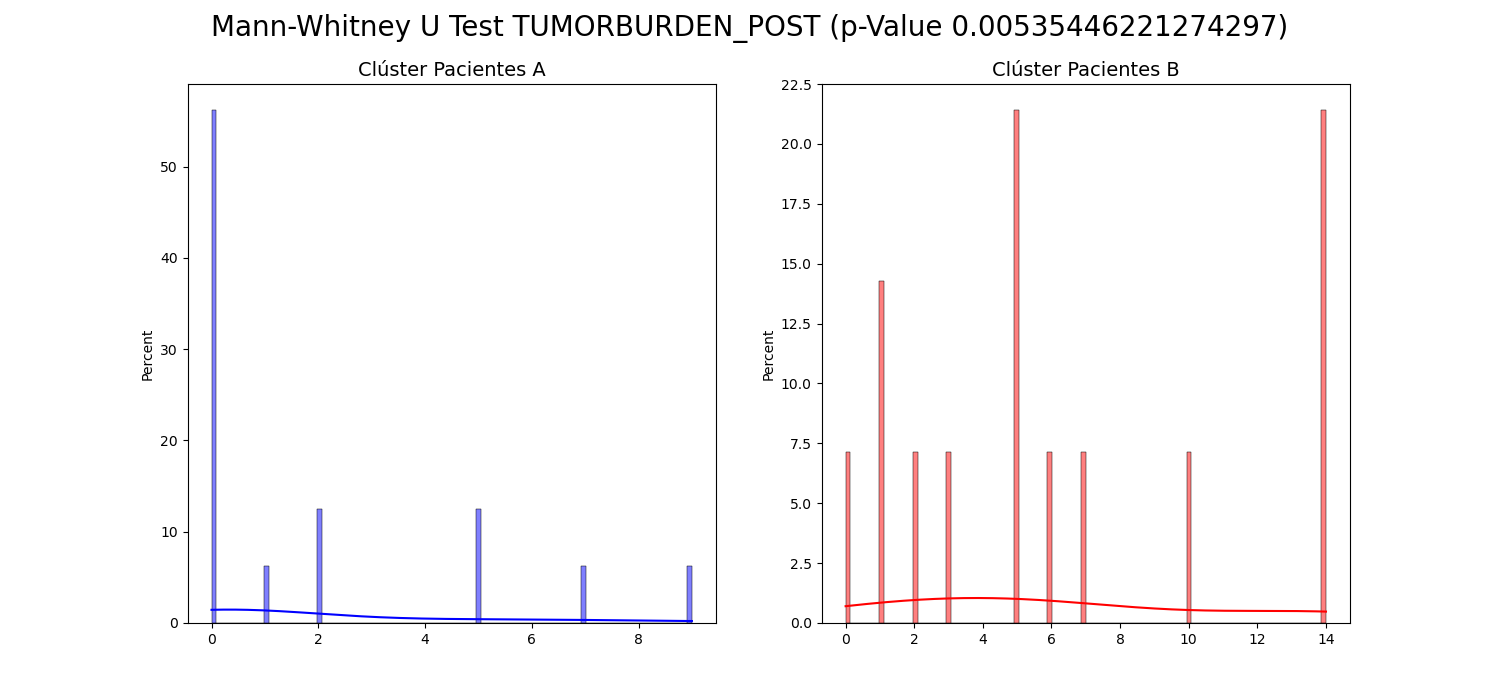


|  |
| --- |

**Figure S****6.** Statistically significant clinical differences were detected between the high and low Qoppa populations. Differences were observed for disease stage, tumor burden, and metastatic burden, both at diagnosis, at the time of sampling, and post-sampling, for all these parameters.﻿​ All these variables showed a non-normal distribution, and in every case, the median value was higher in the high-Qoppa group than in the low-Qoppa group, with the exception of the weighted metastatic burden at diagnosis, where both groups exhibited a median of zero, although with different ranges (0–5 for the low-Qoppa group versus 0–12 for the high-Qoppa group).
