# Supplementary material for: Qoppa as a New Pan-Tumor Synthetic Parameter Derived from Tumor-Associated Biomarkers for Identifying Oncology Patients at High Risk of Metastasis: A Prospective Pilot Study
Source: J Clin Med. 2026 Jan 20;15(2):846. doi: 10.3390/jcm15020846 (PMC12841959; doi:10.3390/jcm15020846)
Supplement: Supplementary file 1 [file jcm-15-00846-s001.zip › DIAZSANTOSetal_Supplementary_TableS1.docx]

Article

Qoppa as a New Pan-Tumor Synthetic Parameter Derived from Tumor-Associated Biomarkers for Identifying Oncology
Patients at High Risk of Metastasis: A Prospective Pilot Study

Javier Diaz-Santos ^1,2,^*, Alba Rodriguez-Valle ^1,2^, Beatriz Berrocal-Gavilan ^1,2^, Olivia Urquizar-Rodriguez ^1,2^
and Silvia Montoro-Garcia ^3^

**Table S1.** Descriptive values of the 11 biomarkers measured through Luminex

| **Range** | **Median** | **Biomarker** |
| --- | --- | --- |
| 173369.59-1053448.09 | 357266.90 | ANGPTL4 (pg/mL) |
| 0-2936798.41 | 855089.07 | Cathepsin D (pg/mL) |
| 0-855.81 | 74.31 | FGF21 (pg/mL) |
| 870.33-42536.41 | 4153.04 | GDF15 (pg/mL) |
| 65.26-587.22 | 228.85 | HGF (pg/mL) |
| 0-2064063.68 | 384502.63 | ICAM1 (pg/mL) |
| 0-766.07 | 54.22 | IL-6 (pg/mL) |
| 0-136.72 | 9.00 | IL-10 (pg/mL) |
| 0-834.77 | 98.54 | IL-18 (pg/mL) |
| 1878.20-63760.20 | 12615.46 | Leptin (pg/mL) |
| 82738.04-4231465.27 | 301577.59 | MPO (pg/mL) |
