# Supplementary material for: Qoppa as a New Pan-Tumor Synthetic Parameter Derived from Tumor-Associated Biomarkers for Identifying Oncology Patients at High Risk of Metastasis: A Prospective Pilot Study
Source: J Clin Med. 2026 Jan 20;15(2):846. doi: 10.3390/jcm15020846 (PMC12841959; doi:10.3390/jcm15020846)
Supplement: Supplementary file 1 [file jcm-15-00846-s001.zip › DIAZSANTOSetal_Supplementary_TableS2.docx]

Article

Qoppa as a New Pan-Tumor Synthetic Parameter Derived from Tumor-Associated Biomarkers for Identifying Oncology
Patients at High Risk of Metastasis: A Prospective Pilot Study

Javier Diaz-Santos ^1,2,^*, Alba Rodriguez-Valle ^1,2^, Beatriz Berrocal-Gavilan ^1,2^, Olivia Urquizar-Rodriguez ^1,2^
and Silvia Montoro-Garcia ^3^

**Table S2.** Main characteristics and performance of the ROC operator curves for the case studies described as supplementary material (figure S2, S3 and S4). Optimism correction with bootstrapping using 1000 bootstraps is applied, showing the corrected area under the curve with its 95% confidence interval. The p-value for comparing the ROC curve against no discriminatory ability (AUC=0.5) using the Mann-Whitney U test is also shown. The optimal cutoff point for each case study, along with the sensitivity and specificity at that point, is also presented.

| **specificity** | **sensitivity** | **cutoff** | **p-value** | **95% Confidence Interval** | **Optimism corrected AUC** | **Original AUC** | **Size (N)** | **Case Study** |
| --- | --- | --- | --- | --- | --- | --- | --- | --- |
| 0.44 | 1.00 | 2.484 | 0.25 | 0.41 - 0.85 | 0.64 | 0.65 | 30 | Qoppa from global analytical parameters (Ϙ_G_) for risk of death (Figure S2.a) |
| 0.52 | 0.86 | 2.07 | 0.13 | 0.46 - 0.89 | 0.69 | 0.69 | 30 | Qoppa from response biomarkers (Ϙ_B_) for risk of death  (Figure S2.b) |
| 0.93 | 1.00 | 5.07 | 0.01 | 0.88 - 1.0 | 0.98 | 0.98 | 18 | Qoppa for risk of death for patients with no metastasis at sample collection (Figure S3.a) |
| 0.63 | 0.0 | 10.61 | 0.73 | 0.10 - 0.78 | 0.44 | 0.44 | 12 | Qoppa for risk of death for patients with metastasis at sample collection (Figure S3.b) |
| 0.69 | 0.8 | 2.69 | 0.15 | 0.41 - 1.0 | 0.72 | 0.72 | 18 | Qoppa from global analytical parameters (Ϙ_G_) for risk of development of metastasis de novo in patients with no metastasis at sample collection (Figure S4.a) |
| 0.69 | 0.5 | 2.25 | 0.08 | 0.5 - 1.0 | 0.76 | 0.77 | 18 | Qoppa from response biomarkers (Ϙ_B_) for risk of development of metastasis de novo in patients with no metastasis at sample collection (Figure S4.b) |
