# Supplementary material for: Qoppa as a New Pan-Tumor Synthetic Parameter Derived from Tumor-Associated Biomarkers for Identifying Oncology Patients at High Risk of Metastasis: A Prospective Pilot Study
Source: J Clin Med. 2026 Jan 20;15(2):846. doi: 10.3390/jcm15020846 (PMC12841959; doi:10.3390/jcm15020846)
Supplement: Supplementary file 1 [file jcm-15-00846-s001.zip › DIAZSANTOSetal_Supplementary_TableS3.docx]

Article

Qoppa as a New Pan-Tumor Synthetic Parameter Derived from Tumor-Associated Biomarkers for Identifying Oncology
Patients at High Risk of Metastasis: A Prospective Pilot Study

Javier Diaz-Santos ^1,2,^*, Alba Rodriguez-Valle ^1,2^, Beatriz Berrocal-Gavilan ^1,2^, Olivia Urquizar-Rodriguez ^1,2^
and Silvia Montoro-Garcia ^3^

**Table S3.** The main characteristics of the study population regarding follow-up time, received treatments before sample collection, and presence of metastases at the time of sample collection are presented. The following abbreviations are introduced: ID - Identification Number, RT - Radiotherapy, ChT - Chemotherapy, ImT - Immunotherapy, and HT - Hormonotherapy. Medians, ranges, and interquartile ranges of the most representative variables are also displayed.

|  |  | **Types of Treatments (0: No received,  1: Received before Sample Collection)** | | | | |  |  |
| --- | --- | --- | --- | --- | --- | --- | --- | --- |
| **Metastasis at Sample Collection** | **Number of Treatments at Sample Collection (N)** | **HT** | **ImT** | **RT** | **ChT** | **Surgery** | **Follow-up Time (days)** | **Patient ID** |
| No | 1 | 0 | 0 | 0 | 0 | 1 | 120 | JER1 |
| No | 4 | 0 | 0 | 1 | 1 | 1 | 51 | JER2 |
| No | 3 | 1 | 1 | 0 | 1 | 1 | 319 | JER3 |
| No | 3 | 0 | 0 | 0 | 1 | 1 | 246 | JER4 |
| No | 3 | 0 | 0 | 1 | 0 | 1 | 203 | JER5 |
| No | 3 | 0 | 0 | 1 | 0 | 1 | 198 | JER6 |
| No | 3 | 0 | 0 | 0 | 1 | 1 | 215 | JER7 |
| No | 2 | 0 | 0 | 0 | 1 | 1 | 215 | JER8 |
| No | 1 | 0 | 0 | 0 | 0 | 1 | 164 | JER9 |
| No | 1 | 0 | 0 | 0 | 0 | 1 | 164 | JER10 |
| No | 2 | 0 | 0 | 0 | 1 | 1 | 391 | JER11 |
| No | 1 | 0 | 0 | 0 | 0 | 1 | 201 | JER12 |
| No | 0 | 0 | 0 | 0 | 0 | 0 | 234 | JER13 |
| No | 0 | 0 | 0 | 0 | 0 | 0 | 215 | JER14 |
| No | 1 | 0 | 0 | 0 | 0 | 1 | 74 | JER15 |
| No | 0 | 0 | 0 | 0 | 0 | 0 | 229 | JER16 |
| No | 1 | 0 | 0 | 1 | 0 | 0 | 166 | JER17 |
| No | 1 | 0 | 0 | 0 | 1 | 0 | 140 | JER18 |
| Yes | 2 | 0 | 0 | 1 | 0 | 1 | 248 | JER19 |
| Yes | 5 | 1 | 1 | 1 | 0 | 1 | 357 | JER20 |
| Yes | 3 | 0 | 0 | 1 | 0 | 1 | 163 | JER21 |
| Yes | 3 | 1 | 1 | 1 | 0 | 1 | 335 | JER22 |
| Yes | 0 | 0 | 0 | 0 | 0 | 0 | 208 | JER23 |
| Yes | 2 | 0 | 0 | 0 | 0 | 0 | 234 | JER24 |
| Yes | 1 | 0 | 0 | 0 | 1 | 0 | 157 | JER25 |
| Yes | 1 | 0 | 0 | 0 | 0 | 1 | 214 | JER26 |
| Yes | 0 | 0 | 0 | 0 | 0 | 0 | 11 | JER27 |
| Yes | 3 | 0 | 0 | 1 | 1 | 1 | 80 | JER28 |
| Yes | 4 | 1 | 1 | 1 | 1 | 0 | 51 | JER29 |
| Yes | 2 | 0 | 0 | 0 | 1 | 0 | 130 | JER30 |
| No:Yes (18:12) | 56 | 5 | 4 | 10 | 11 | 19 | 5733 | **Total** |
|  | 2 | 0 | 0 | 0 | 0 | 1 | 202 | **Median** |
|  | 0-5 | 0-1 | 0-1 | 0-1 | 0-1 | 0-1 | 11-391 | **Range** |
|  | 1-3 |  |  |  |  |  | 144.25-232.75 | **Interquartile Range** |
