# Supplementary material for: Qoppa as a New Pan-Tumor Synthetic Parameter Derived from Tumor-Associated Biomarkers for Identifying Oncology Patients at High Risk of Metastasis: A Prospective Pilot Study
Source: J Clin Med. 2026 Jan 20;15(2):846. doi: 10.3390/jcm15020846 (PMC12841959; doi:10.3390/jcm15020846)
Supplement: Supplementary file 1 [file jcm-15-00846-s001.zip › DIAZSANTOSetal_Supplementary_TableS4.docx]

Article

Qoppa as a New Pan-Tumor Synthetic Parameter Derived from Tumor-Associated Biomarkers for Identifying Oncology
Patients at High Risk of Metastasis: A Prospective Pilot Study

Javier Diaz-Santos ^1,2,^*, Alba Rodriguez-Valle ^1,2^, Beatriz Berrocal-Gavilan ^1,2^, Olivia Urquizar-Rodriguez ^1,2^
and Silvia Montoro-Garcia ^3^

Disease burden was characterized using two complementary composite indices: weighted metastatic burden was calculated as the aggregate count of radiologically visible metastatic lesions across all anatomic sites, with scores ranging from 0 to 4 for each location (based on the number of lesions, where 4 denoted four or more lesions in total). The overall tumor burden was derived by adding the weighted metastatic burden score plus one point each for the presence of primary tumor and locoregional lymph node involvement. These values are shown in Table S4 presented below.

Both the weighted metastatic burden and total tumor burden were quantified by a single investigator in duplicate. Parameters were first derived from direct review of the most recent pre-sample collection imaging studies per patient. Findings were then independently validated by recalculating parameters using electronic health record data and corresponding radiology reports by the same investigator.

**Table S4.** Summary of metastatic sites and number of metastases (Met.) per location, as well as tumor and lymph node involvement at sample collection, and calculation of weighted metastatic burden and overall tumor burden.

| **Number of Metastases in each Location** | | | | | | |  | **(0: Non present, 1: Present)** | |  |  |  |
| --- | --- | --- | --- | --- | --- | --- | --- | --- | --- | --- | --- | --- |
| **Skin** | **Ren.** | **Perit.** | **Brain** | **Lung** | **Liver** | **Bone** | **Weighted Met. Burden** | **Lymph Nodes Involvement at Sample Collection** | **Tumor at Sample Collection** | **Overall Tumor Burden** | **Met. at Sample Collection** | **Patient ID** |
| 0 | 0 | 0 | 0 | 0 | 0 | 0 | 0 | 0 | 0 | 0 | No | JER1 |
| 0 | 0 | 0 | 0 | 0 | 0 | 0 | 0 | 0 | 0 | 0 | No | JER2 |
| 0 | 0 | 0 | 0 | 0 | 0 | 0 | 0 | 0 | 0 | 0 | No | JER3 |
| 0 | 0 | 0 | 0 | 0 | 0 | 0 | 0 | 0 | 0 | 0 | No | JER4 |
| 0 | 0 | 0 | 0 | 0 | 0 | 0 | 0 | 0 | 0 | 0 | No | JER5 |
| 0 | 0 | 0 | 0 | 0 | 0 | 0 | 0 | 0 | 0 | 0 | No | JER6 |
| 0 | 0 | 0 | 0 | 0 | 0 | 0 | 0 | 0 | 0 | 0 | No | JER7 |
| 0 | 0 | 0 | 0 | 0 | 0 | 0 | 0 | 0 | 0 | 0 | No | JER8 |
| 0 | 0 | 0 | 0 | 0 | 0 | 0 | 0 | 0 | 0 | 0 | No | JER9 |
| 0 | 0 | 0 | 0 | 0 | 0 | 0 | 0 | 0 | 0 | 0 | No | JER10 |
| 0 | 0 | 0 | 0 | 0 | 0 | 0 | 0 | 0 | 0 | 0 | No | JER11 |
| 0 | 0 | 0 | 0 | 0 | 0 | 0 | 0 | 0 | 0 | 0 | No | JER12 |
| 0 | 0 | 0 | 0 | 0 | 0 | 0 | 0 | 1 | 1 | 2 | No | JER13 |
| 0 | 0 | 0 | 0 | 0 | 0 | 0 | 0 | 1 | 1 | 2 | No | JER14 |
| 0 | 0 | 0 | 0 | 0 | 0 | 0 | 0 | 0 | 1 | 1 | No | JER15 |
| 0 | 0 | 0 | 0 | 0 | 0 | 0 | 0 | 1 | 1 | 2 | No | JER16 |
| 0 | 0 | 0 | 0 | 0 | 0 | 0 | 0 | 1 | 1 | 2 | No | JER17 |
| 0 | 0 | 0 | 0 | 0 | 0 | 0 | 0 | 1 | 1 | 2 | No | JER18 |
| 0 | 0 | 0 | 0 | 0 | 0 | >4 | 4 | 0 | 1 | 5 | Yes | JER19 |
| 0 | 2 | 1 | 0 | 1 | 0 | 0 | 4 | 0 | 0 | 4 | Yes | JER20 |
| 0 | 0 | 0 | 0 | >4 | 0 | 0 | 4 | 0 | 1 | 5 | Yes | JER21 |
| 0 | 0 | 0 | 1 | 0 | 0 | 0 | 1 | 0 | 0 | 1 | Yes | JER22 |
| 0 | 0 | 0 | 0 | 0 | >4 | 1 | 5 | 1 | 1 | 7 | Yes | JER23 |
| 0 | 0 | 0 | 0 | 0 | 0 | >4 | 4 | 0 | 1 | 5 | Yes | JER24 |
| 0 | 0 | 0 | 0 | >4 | >4 | >4 | 12 | 1 | 1 | 14 | Yes | JER25 |
| 0 | 0 | >4 | 0 | 0 | 0 | 0 | 4 | 0 | 1 | 5 | Yes | JER26 |
| 0 | 0 | >4 | 0 | 0 | >4 | 0 | 8 | 1 | 1 | 10 | Yes | JER27 |
| 1 | 0 | 0 | 0 | >4 | 0 | 0 | 5 | 1 | 1 | 7 | Yes | JER28 |
| 0 | 0 | 0 | 0 | >4 | 1 | >4 | 9 | 1 | 1 | 11 | Yes | JER29 |
| 0 | 0 | 0 | 0 | >4 | >4 | >4 | 12 | 1 | 1 | 14 | Yes | JER30 |
| 1 | 2 | 9 | 1 | 21 | 17 | 21 | 72 | 11 | 16 | 99 | No:Yes (18:12) | **Total** |
| 0 | 0 | 0 | 0 | 0 | 0 | 0 | 0 | 0 | 1 | 2 |  | **Median** |
|  |  |  |  |  |  |  | 0-12 |  |  | 0-14 |  | **Range** |
|  |  |  |  |  |  |  | 0-4 |  |  | 0-5 |  | **Interquart. Range** |
