# Supplementary material for: Qoppa as a New Pan-Tumor Synthetic Parameter Derived from Tumor-Associated Biomarkers for Identifying Oncology Patients at High Risk of Metastasis: A Prospective Pilot Study
Source: J Clin Med. 2026 Jan 20;15(2):846. doi: 10.3390/jcm15020846 (PMC12841959; doi:10.3390/jcm15020846)
Supplement: Supplementary file 1 [file jcm-15-00846-s001.zip › DIAZSANTOSetal_Supplementary_FigureS1.pdf]

Journal of
Clinical Medicine

MDPI

Article

Qoppa as a New Pan-Tumor Synthetic Parameter Derived from Tumor-Associated Biomarkers for Identifying Oncology Patients at High Risk of Metastasis: A Prospective Pilot Study

Javier Diaz-Santos [1,2,*], Alba Rodriguez-Valle [1,2], Beatriz Berrocal-Gavilan [1,2], Olivia Urquizar-Rodriguez [1,2] and Silvia Montoro-Garcia [3]

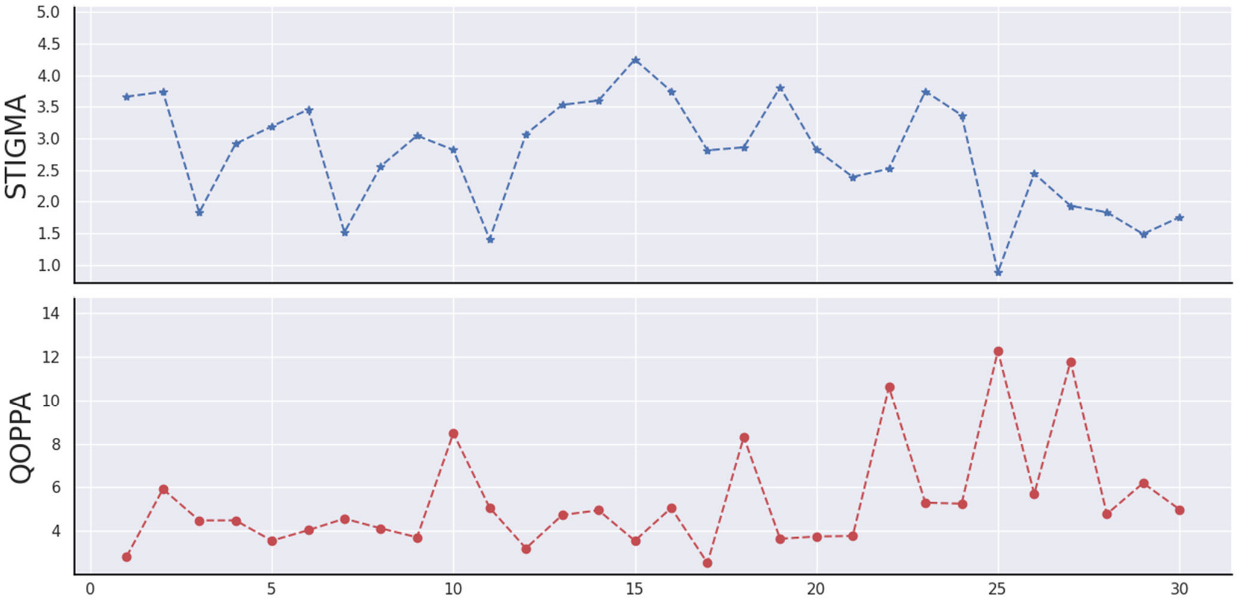

Figure S1. Profile of synthetic variable values of clusters Stigma (Ϛ) and Qoppa (Ϙ)
