# Supplementary material for: Qoppa as a New Pan-Tumor Synthetic Parameter Derived from Tumor-Associated Biomarkers for Identifying Oncology Patients at High Risk of Metastasis: A Prospective Pilot Study
Source: J Clin Med. 2026 Jan 20;15(2):846. doi: 10.3390/jcm15020846 (PMC12841959; doi:10.3390/jcm15020846)
Supplement: Supplementary file 1 [file jcm-15-00846-s001.zip › DIAZSANTOSetal_Supplementary_FigureS4.pdf]

Journal of
Clinical Medicine

MDPI

Article

Qoppa as a New Pan-Tumor Synthetic Parameter Derived from Tumor-Associated Biomarkers for Identifying Oncology Patients at High Risk of Metastasis: A Prospective Pilot Study

Javier Diaz-Santos [1,2,*], Alba Rodriguez-Valle [1,2], Beatriz Berrocal-Gavilan [1,2], Olivia Urquizar-Rodriguez [1,2] and Silvia Montoro-Garcia [3]

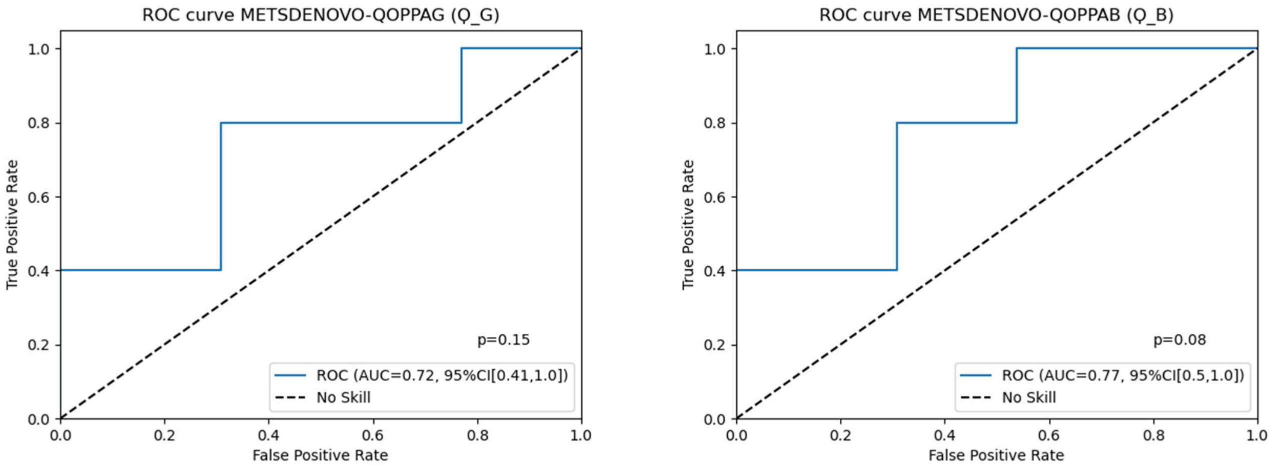

(**a**) (**b**)

Figure S4. ROC curve analysis study of the role of the component of Qoppa from global analytical parameters (Ϙ_G, panel a) and from response biomarkers (Ϙ_B, panel b) as a classifier of the risk of development of metastasis de novo in patients with no metastasis at sample collection. For each case, the 95% confidence interval and p-value for comparing the ROC curve against no discriminatory ability (AUC=0.5) using the Mann-Whitney U test are also shown.
