# Supplementary material for: Qoppa as a New Pan-Tumor Synthetic Parameter Derived from Tumor-Associated Biomarkers for Identifying Oncology Patients at High Risk of Metastasis: A Prospective Pilot Study
Source: J Clin Med. 2026 Jan 20;15(2):846. doi: 10.3390/jcm15020846 (PMC12841959; doi:10.3390/jcm15020846)
Supplement: Supplementary file 1 [file jcm-15-00846-s001.zip › DIAZSANTOSetal_Supplementary_FigureS5.pdf]

Journal of Clinical Medicine

Article

Qoppa as a New Pan-Tumor Synthetic Parameter Derived from Tumor-Associated Biomarkers for Identifying Oncology Patients at High Risk of Metastasis: A Prospective Pilot Study

Javier Diaz-Santos [1,2,*], Alba Rodriguez-Valle [1,2], Beatriz Berrocal-Gavilan [1,2], Olivia Urquizar-Rodriguez [1,2] and Silvia Montoro-Garcia [3]

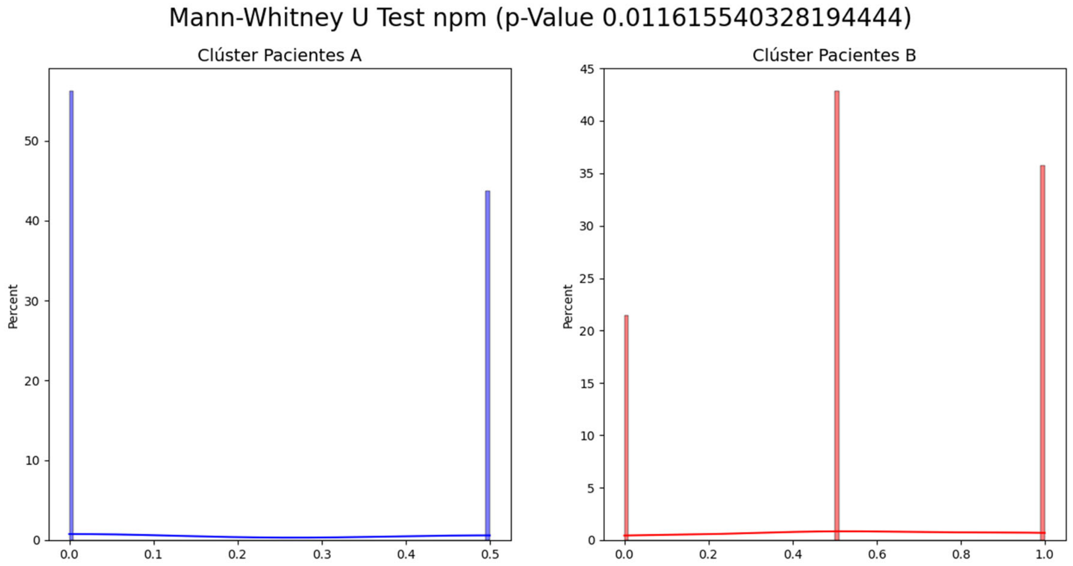

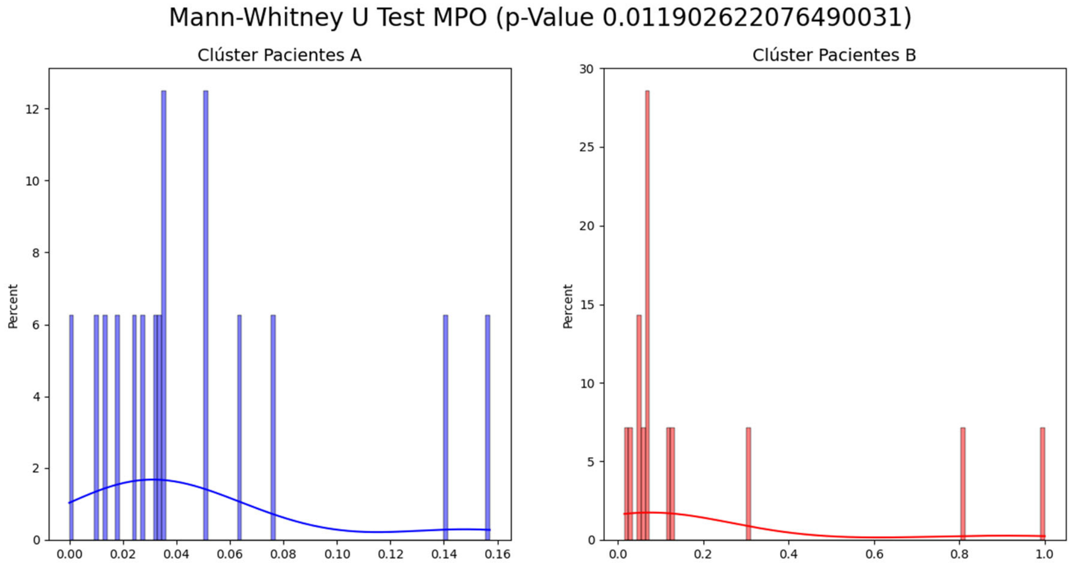

 https://doi.org/10.3390/jcm15020846

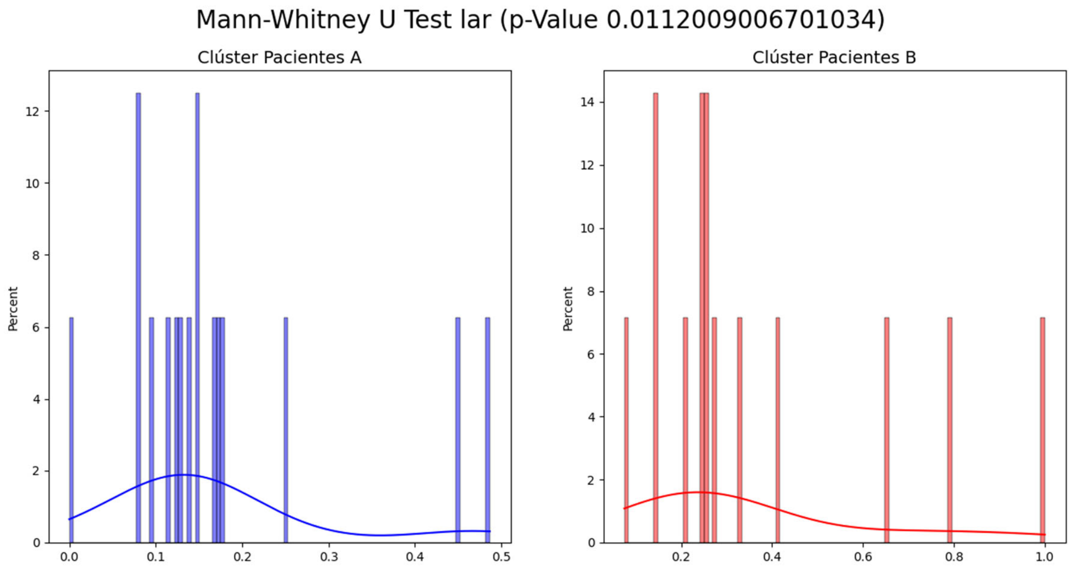

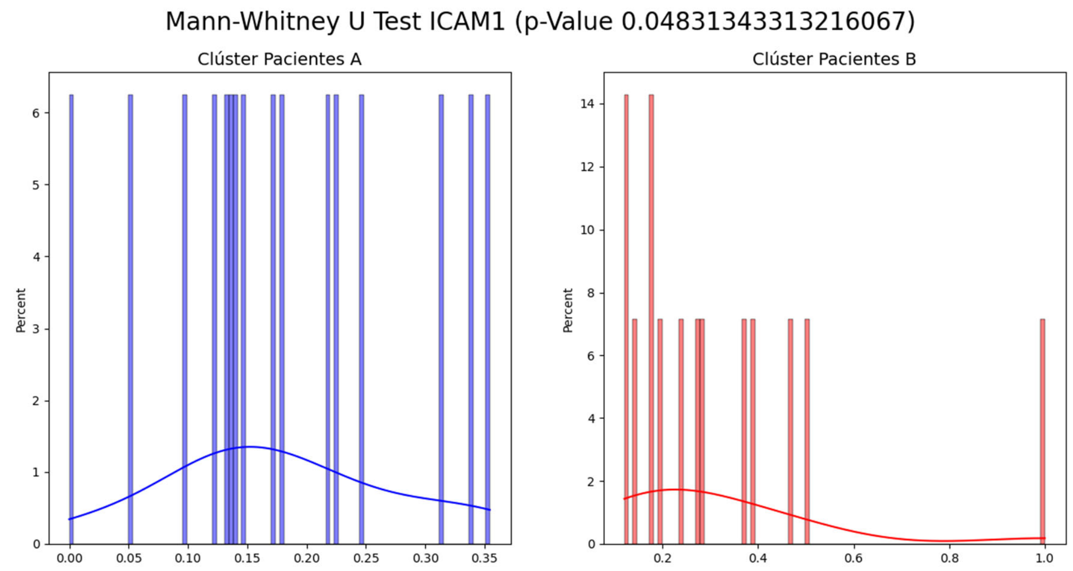

https://doi.org/10.3390/jcm15020846

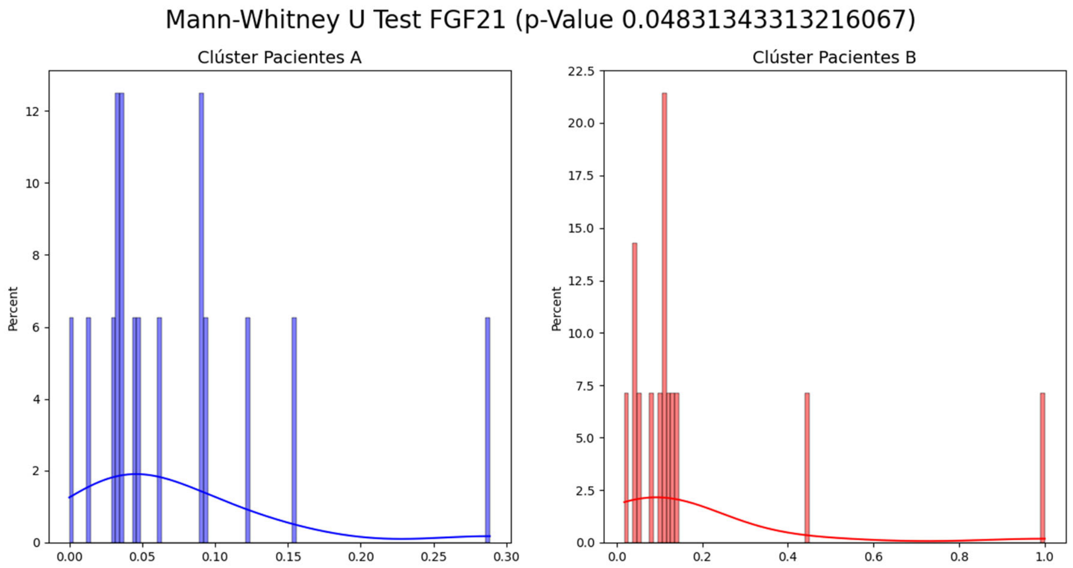

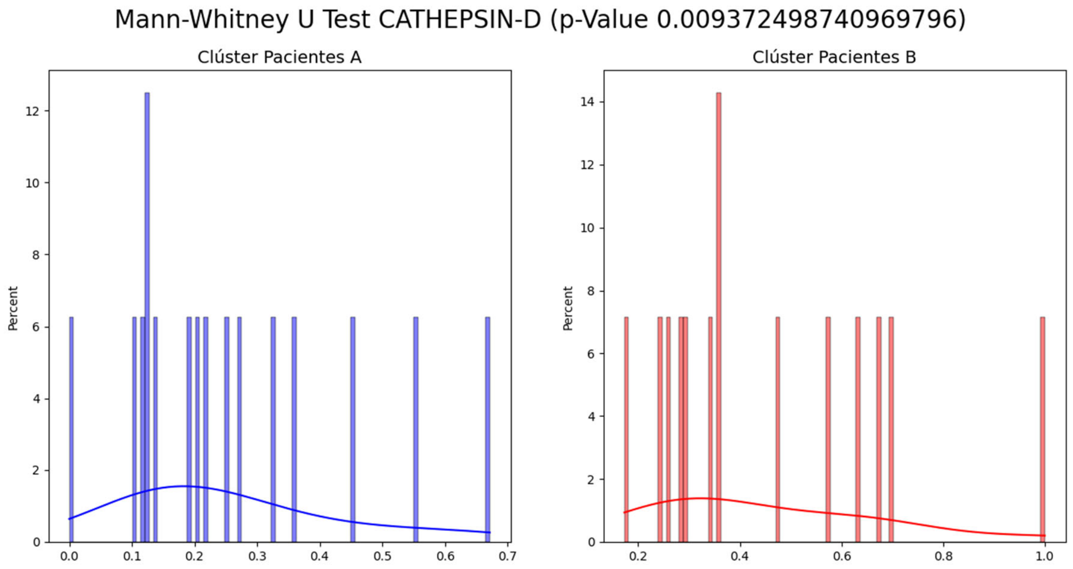

https://doi.org/10.3390/jcm15020846

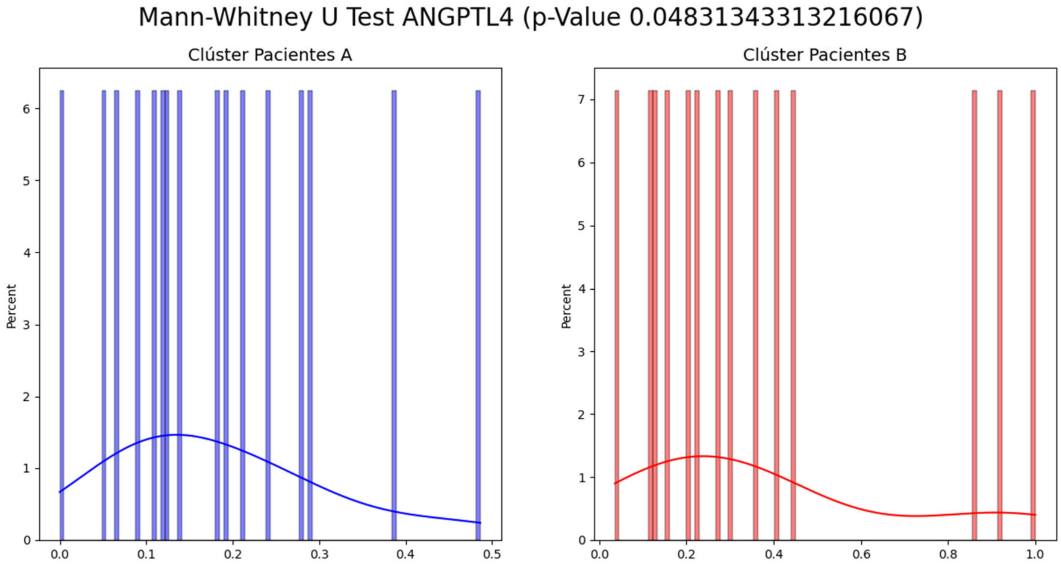

Figure S5. Statistically significant analytical differences were detected between the high and low Qoppa populations. Differences were observed for ANGPTL4, CATHEPSIN-D, FGF21, ICAM1, MPO, lar, and npm. The aliases for global laboratory parameters are shown written in lowercase and in uppercase for response biomarkers. All these variables showed a non-normal distribution, and in every case, the median value was higher in the high-Qoppa group than in the low-Qoppa group.
