# Supplementary material for: Qoppa as a New Pan-Tumor Synthetic Parameter Derived from Tumor-Associated Biomarkers for Identifying Oncology Patients at High Risk of Metastasis: A Prospective Pilot Study
Source: J Clin Med. 2026 Jan 20;15(2):846. doi: 10.3390/jcm15020846 (PMC12841959; doi:10.3390/jcm15020846)
Supplement: Supplementary file 1 [file jcm-15-00846-s001.zip › DIAZSANTOSetal_Supplementary_TableS1.pdf]

Journal of
Clinical Medicine

MDPI

Article

Qoppa as a New Pan-Tumor Synthetic Parameter Derived from Tumor-Associated Biomarkers for Identifying Oncology Patients at High Risk of Metastasis: A Prospective Pilot Study

Javier Diaz-Santos [1,2,*], Alba Rodriguez-Valle [1,2], Beatriz Berrocal-Gavilan [1,2], Olivia Urquizar-Rodriguez [1,2] and Silvia Montoro-Garcia [3]

Table S1. Descriptive values of the 11 biomarkers measured through Luminex

Biomarker	Median	Range
ANGPTL4 (pg/mL)	357266.90	173369.59-1053448.09
Cathepsin D (pg/mL)	855089.07	0-2936798.41
FGF21 (pg/mL)	74.31	0-855.81
GDF15 (pg/mL)	4153.04	870.33-42536.41
HGF (pg/mL)	228.85	65.26-587.22
ICAM1 (pg/mL)	384502.63	0-2064063.68
IL-6 (pg/mL)	54.22	0-766.07
IL-10 (pg/mL)	9.00	0-136.72
IL-18 (pg/mL)	98.54	0-834.77
Leptin (pg/mL)	12615.46	1878.20-63760.20
MPO (pg/mL)	301577.59	82738.04-4231465.27

 https://doi.org/10.3390/jcm15020846
