# Supplementary material for: Qoppa as a New Pan-Tumor Synthetic Parameter Derived from Tumor-Associated Biomarkers for Identifying Oncology Patients at High Risk of Metastasis: A Prospective Pilot Study
Source: J Clin Med. 2026 Jan 20;15(2):846. doi: 10.3390/jcm15020846 (PMC12841959; doi:10.3390/jcm15020846)
Supplement: Supplementary file 1 [file jcm-15-00846-s001.zip › DIAZSANTOSetal_Supplementary_TableS3.pdf]

Journal of Clinical Medicine

Article

Qoppa as a New Pan-Tumor Synthetic Parameter Derived from Tumor-Associated Biomarkers for Identifying Oncology Patients at High Risk of Metastasis: A Prospective Pilot Study

Javier Diaz-Santos [1,2,*], Alba Rodriguez-Valle [1,2], Beatriz Berrocal-Gavilan [1,2], Olivia Urquizar-Rodriguez [1,2] and Silvia Montoro-Garcia [3]

Table S3. The main characteristics of the study population regarding follow-up time, received treatments before sample collection, and presence of metastases at the time of sample collection are presented. The following abbreviations are introduced: ID - Identification Number, RT - Radiotherapy, ChT - Chemotherapy, ImT - Immunotherapy, and HT - Hormonotherapy. Medians, ranges, and interquartile ranges of the most representative variables are also displayed.

Patient ID	Follow-up Time (days)	Types of Treatments (0: No received, 1: Received before Sample Collection)					Number of Treatments at Sample Collection (N)	Metastasis at Sample Collection
		Surgery	ChT	RT	ImT	HT		
JER1	120	1	0	0	0	0	1	No
JER2	51	1	1	1	0	0	4	No
JER3	319	1	1	0	1	1	3	No
JER4	246	1	1	0	0	0	3	No
JER5	203	1	0	1	0	0	3	No
JER6	198	1	0	1	0	0	3	No
JER7	215	1	1	0	0	0	3	No
JER8	215	1	1	0	0	0	2	No
JER9	164	1	0	0	0	0	1	No
JER10	164	1	0	0	0	0	1	No
JER11	391	1	1	0	0	0	2	No
JER12	201	1	0	0	0	0	1	No
JER13	234	0	0	0	0	0	0	No
JER14	215	0	0	0	0	0	0	No
JER15	74	1	0	0	0	0	1	No
JER16	229	0	0	0	0	0	0	No
JER17	166	0	0	1	0	0	1	No
JER18	140	0	1	0	0	0	1	No
JER19	248	1	0	1	0	0	2	Yes
JER20	357	1	0	1	1	1	5	Yes
JER21	163	1	0	1	0	0	3	Yes
JER22	335	1	0	1	1	1	3	Yes
JER23	208	0	0	0	0	0	0	Yes
JER24	234	0	0	0	0	0	2	Yes

JER25	157	0	1	0	0	0	1	Yes
JER26	214	1	0	0	0	0	1	Yes
JER27	11	0	0	0	0	0	0	Yes
JER28	80	1	1	1	0	0	3	Yes
JER29	51	0	1	1	1	1	4	Yes
JER30	130	0	1	0	0	0	2	Yes
**Total**	5733	19	11	10	4	5	56	No:Yes (18:12)
**Median**	202	1	0	0	0	0	2	
**Range**	11-391	0-1	0-1	0-1	0-1	0-1	0-5	
**Interquartile Range**	144.25-232.75						1-3	

https://doi.org/10.3390/jcm15020846
