# Supplementary material for: Qoppa as a New Pan-Tumor Synthetic Parameter Derived from Tumor-Associated Biomarkers for Identifying Oncology Patients at High Risk of Metastasis: A Prospective Pilot Study
Source: J Clin Med. 2026 Jan 20;15(2):846. doi: 10.3390/jcm15020846 (PMC12841959; doi:10.3390/jcm15020846)
Supplement: Supplementary file 1 [file jcm-15-00846-s001.zip › DIAZSANTOSetal_Supplementary_TableS4.pdf]

Journal of *Clinical Medicine*

MDPI

Article

Qoppa as a New Pan-Tumor Synthetic Parameter Derived from Tumor-Associated Biomarkers for Identifying Oncology Patients at High Risk of Metastasis: A Prospective Pilot Study

Javier Diaz-Santos [1,2,*], Alba Rodriguez-Valle [1,2], Beatriz Berrocal-Gavilan [1,2], Olivia Urquizar-Rodriguez [1,2] and Silvia Montoro-Garcia [3]

Disease burden was characterized using two complementary composite indices: weighted metastatic burden was calculated as the aggregate count of radiologically visible metastatic lesions across all anatomic sites, with scores ranging from 0 to 4 for each location (based on the number of lesions, where 4 denoted four or more lesions in total). The overall tumor burden was derived by adding the weighted metastatic burden score plus one point each for the presence of primary tumor and locoregional lymph node involvement. These values are shown in Table S4 presented below.

Both the weighted metastatic burden and total tumor burden were quantified by a single investigator in duplicate. Parameters were first derived from direct review of the most recent pre-sample collection imaging studies per patient. Findings were then independently validated by recalculating parameters using electronic health record data and corresponding radiology reports by the same investigator.

Table S4. Summary of metastatic sites and number of metastases (Met.) per location, as well as tumor and lymph node involvement at sample collection, and calculation of weighted metastatic burden and overall tumor burden.

			(0: Non present, 1: Present)			Number of Metastases in each Location						
**Patient ID**	**Met. at Sample Collection**	**Overall Tumor Burden**	**Tumor at Sample Collection**	**Lymph Nodes Involvement at Sample Collection**	**Weighted Met. Burden**	**Bone**	**Liver**	**Lung**	**Brain**	**Perit.**	**Ren.**	**Skin**
JER1	No	0	0	0	0	0	0	0	0	0	0	0
JER2	No	0	0	0	0	0	0	0	0	0	0	0
JER3	No	0	0	0	0	0	0	0	0	0	0	0
JER4	No	0	0	0	0	0	0	0	0	0	0	0
JER5	No	0	0	0	0	0	0	0	0	0	0	0
JER6	No	0	0	0	0	0	0	0	0	0	0	0
JER7	No	0	0	0	0	0	0	0	0	0	0	0
JER8	No	0	0	0	0	0	0	0	0	0	0	0
JER9	No	0	0	0	0	0	0	0	0	0	0	0
JER10	No	0	0	0	0	0	0	0	0	0	0	0
JER11	No	0	0	0	0	0	0	0	0	0	0	0
JER12	No	0	0	0	0	0	0	0	0	0	0	0
JER13	No	2	1	1	0	0	0	0	0	0	0	0
JER14	No	2	1	1	0	0	0	0	0	0	0	0
JER15	No	1	1	0	0	0	0	0	0	0	0	0

 https://doi.org/10.3390/jcm15020846

JER16	No	2	1	1	0	0	0	0	0	0	0	0
JER17	No	2	1	1	0	0	0	0	0	0	0	0
JER18	No	2	1	1	0	0	0	0	0	0	0	0
JER19	Yes	5	1	0	4	>4	0	0	0	0	0	0
JER20	Yes	4	0	0	4	0	0	1	0	1	2	0
JER21	Yes	5	1	0	4	0	0	>4	0	0	0	0
JER22	Yes	1	0	0	1	0	0	0	1	0	0	0
JER23	Yes	7	1	1	5	1	>4	0	0	0	0	0
JER24	Yes	5	1	0	4	>4	0	0	0	0	0	0
JER25	Yes	14	1	1	12	>4	>4	>4	0	0	0	0
JER26	Yes	5	1	0	4	0	0	0	0	>4	0	0
JER27	Yes	10	1	1	8	0	>4	0	0	>4	0	0
JER28	Yes	7	1	1	5	0	0	>4	0	0	0	1
JER29	Yes	11	1	1	9	>4	1	>4	0	0	0	0
JER30	Yes	14	1	1	12	>4	>4	>4	0	0	0	0
**Total**	No:Yes (18:12)	99	16	11	72	21	17	21	1	9	2	1
**Median**		2	1	0	0	0	0	0	0	0	0	0
**Range**		0-14			0-12							
**Interquart. Range**		0-5			0-4							
